# Editorial Note: *In Vitro* and *In Vivo* Antitumor Activity of [Pt(*O,O′*-acac)(γ-acac)(DMS)] in Malignant Pleural Mesothelioma

**DOI:** 10.1371/journal.pone.0353915

**Published:** 2026-07-16

**Authors:** The PLOS One Editors

After this article [1] was published, concerns were raised regarding Figs 2-5. Specifically,

- The following panels in Figs 3-5 appear to overlap with one another and/or with a later article published by the same research group:
  - Lanes 2-4 of the Mitochondrial fractions Cyt.C panel of Fig 3B and lanes 2-4 of the Ptac2S Cyt.c panel of Fig 5D.
  - Lanes 1-2 of the PARP-1 panel of Fig 4F, lanes 1-2 of the Ptac2S PARP-1 panel of Fig 5D, and lanes 1-2 of the PARP-1 panel in Fig 5C of [2].
- The following panels in Figs 2, 3 and 5 appear to contain overlapping regions within the same panel:
  - The background between lanes 1-2, and 2-3 of the Cisplatin Casp-3 panel of Fig 2D.
  - Lanes 2 and 5 of the Cisplatin β-actin panel of Fig 2D.
  - Lanes 1 and 2 of the Cytosolic fractions β-actin panel of Fig 3B.
  - Lanes 1-2 and 5-6 of the p-p38MAPK Cisplatin panel of Fig 5A.
- The [Pt(*O*,*O′*-acac)(γ-acac)(DMS)] p38MAPK panel in Fig 5A appears to overlap with the ZL55 p-p38MAPK panel in Fig 4A of [2].

The corresponding author provided the original, underlying images for all panels of concern. They further stated that the [Pt(*O*,*O′*-acac)(γ-acac)(DMS)] p38MAPK panel in Fig 5A of [1] was intentionally reused in the ZL55 p-p38MAPK panel in Fig 4A of [2], as the panels originated from the same time-course experiment examining the activation of p38MAPK in ZL55 cells following treatment with the platinum complex.

Following editorial review of the original, underlying images (provided here as S1 File), PLOS considers all the above concerns resolved.

In addition, contrary to the declaration in the Data Availability statement, the original raw data files supporting the article’s results were not provided with the article. The authors have provided the data as S2 File. With this notice, all relevant data are now provided.

## Supporting information

S1 File Original blots underlying panels of concern with Figs 2–5. (ZIP)

S2 File Original quantitative data underlying all Figs. (PDF)

## References

[1] Muscella A, Vetrugno C, Cossa LG, Antonaci G, De Nuccio F, De Pascali SA, et al. *In Vitro* and *In Vivo* Antitumor Activity of [Pt(*O*,*O′*-acac)(γ-acac)(DMS)] in Malignant Pleural Mesothelioma. PLoS One. 2016;11(11):e0165154. doi: 10.1371/journal.pone.0165154 27806086 PMC5091852

[2] Muscella A, Vetrugno C, Cossa LG, Antonaci G, Barca A, De Pascali SA, et al. Apoptosis by [Pt(*O*,*O′*-acac)(γ-acac)(DMS)] requires PKC-δ mediated p53 activation in malignant pleural mesothelioma. PLoS One. 2017;12(7):e0181114. doi: 10.1371/journal.pone.0181114 28704484 PMC5507537

