# Supplementary figures and images for: Editorial Note: In Vitro and In Vivo Antitumor Activity of [Pt(O,O′-acac)(γ-acac)(DMS)] in Malignant Pleural Mesothelioma
Source: PLoS One. 2026 Jul 16;21(7):e0353915. doi: 10.1371/journal.pone.0353915 (PMC13375038; doi:10.1371/journal.pone.0353915)

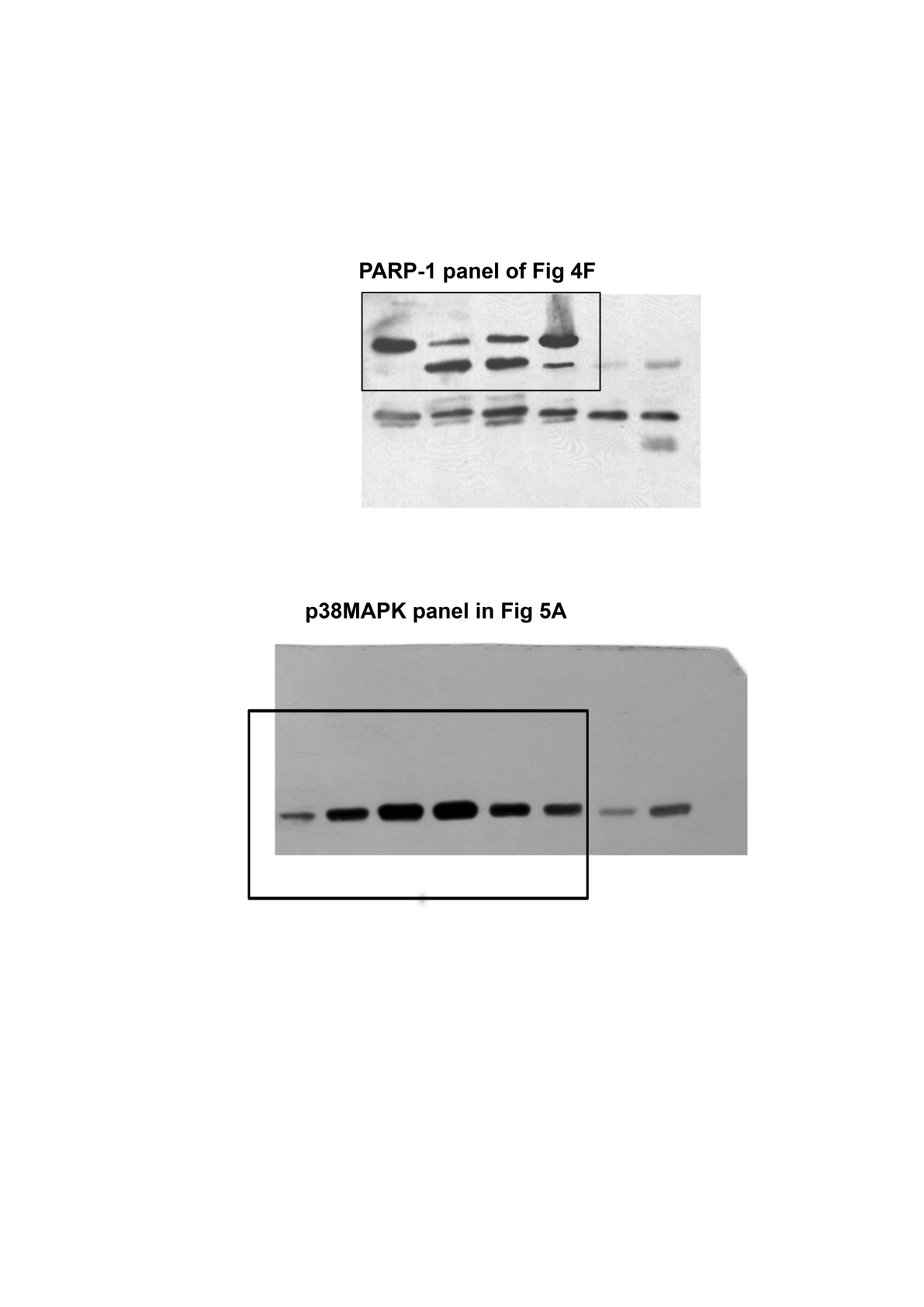

Supplement: S1 File — (ZIP) [file pone.0353915.s001.zip › S1 - Original blots underlying panels within Figs 2-5/S1A.tif]

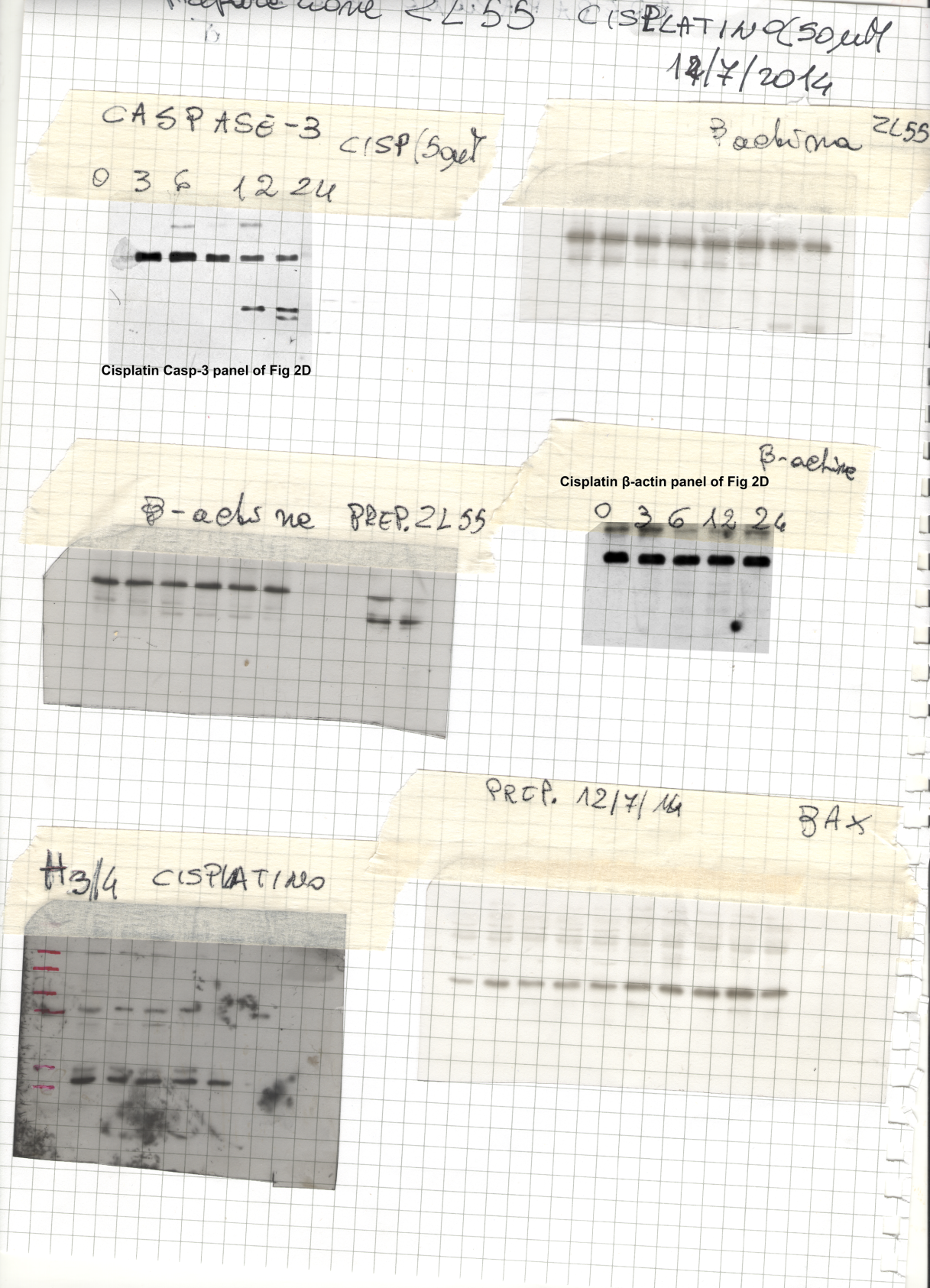

Supplement: S1 File — (ZIP) [file pone.0353915.s001.zip › S1 - Original blots underlying panels within Figs 2-5/S1B.tif]

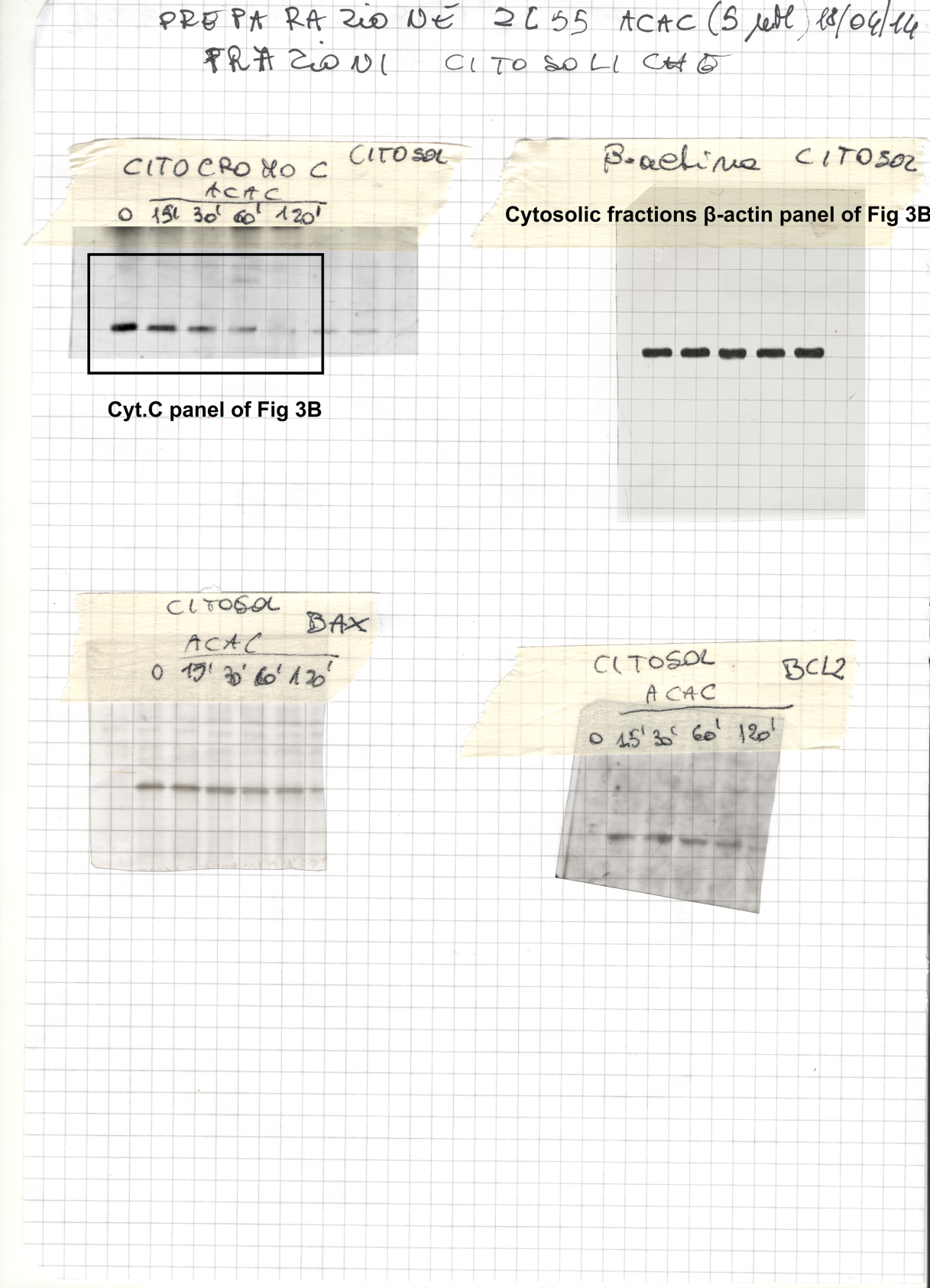

Supplement: S1 File — (ZIP) [file pone.0353915.s001.zip › S1 - Original blots underlying panels within Figs 2-5/S1C.tif]

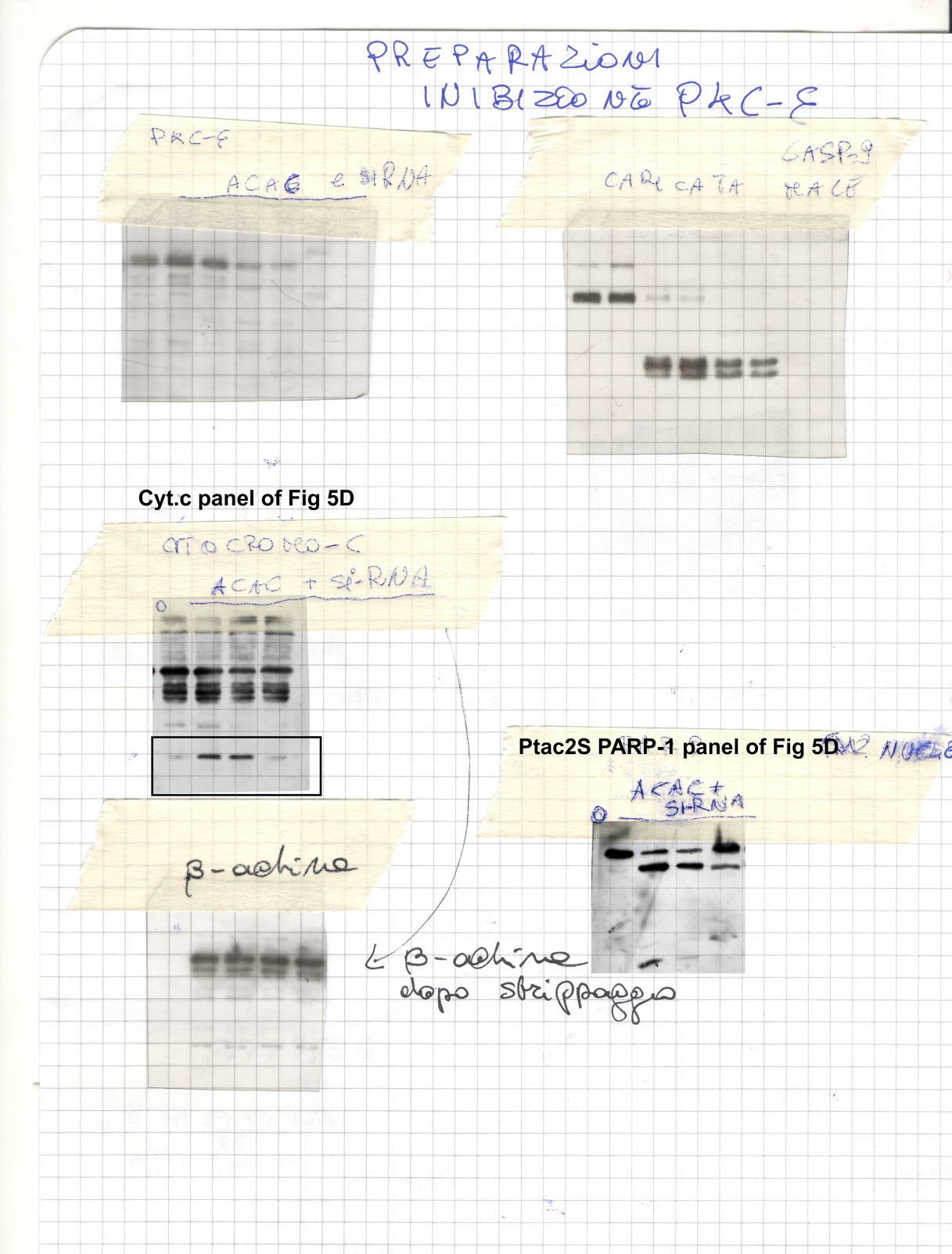

Supplement: S1 File — (ZIP) [file pone.0353915.s001.zip › S1 - Original blots underlying panels within Figs 2-5/S1D.tif]
