# Supplementary material for: Editorial Note: In Vitro and In Vivo Antitumor Activity of [Pt(O,O′-acac)(γ-acac)(DMS)] in Malignant Pleural Mesothelioma
Source: PLoS One. 2026 Jul 16;21(7):e0353915. doi: 10.1371/journal.pone.0353915 (PMC13375038; doi:10.1371/journal.pone.0353915)
Supplement: S2 File — (PDF) [file pone.0353915.s002.pdf]

Figure 1: Cell Viability and Clonogenicity e ciclo cellulare

12h Viability (% of control)

Dose (µmol L^{-1})	Cis_E1	Cis_E2	Cis_E3	Cis_E4	Cis_E5	Cis_E6	Media	S.D.	Pt_E1	Pt_E2	Pt_E3	Pt_E4	Pt_E5	Pt_E6	Media	S.D.
0	100,0	100,0	100,0	100,0	100,0	100,0	100,0	0,0	100,0	100,0	100,0	100,0	100,0	100,0	100,0	0,0
1	98,2	101,5	95,3	99,1	96,4	101,5	98,7	2,3	63,5	72,1	67,4	69,8	65,2	68,0	67,7	2,8
10	89,1	94,6	90,3	92,5	88,7	92,8	91,3	2,1	36,2	42,8	38,0	40,5	37,1	39,4	39,0	2,2
100	53,4	56,1	49,5	55,2	51,0	54,8	53,3	2,4	27,1	35,4	28,5	32,3	29,1	31,6	30,7	2,8
200	48,2	54,0	48,8	52,1	47,5	51,4	50,3	2,3	22,4	28,9	24,7	26,8	23,1	26,1	25,3	2,2

24h

Dose (µmol L^{-1})	Cis_E1	Cis_E2	Cis_E3	Cis_E4	Cis_E5	Cis_E6	Media	S.D.	Pt_E1	Pt_E2	Pt_E3	Pt_E4	Pt_E5	Pt_E6	Media	S.D.
0	100,0	100,0	100,0	100,0	100,0	100,0	100,0	0,0	100,0	100,0	100,0	100,0	100,0	100,0	100,0	0,0
1	94,1	99,8	95,1	98,2	93,9	96,9	96,3	2,2	61,2	70,4	64,3	67,1	62,5	64,5	65,0	3,0
10	61,2	68,4	64,4	66,8	62,1	65,1	64,7	2,5	27,3	34,1	29,6	32,2	28,4	30,4	30,3	2,3
100	32,1	37,9	34,0	36,5	32,4	35,1	34,7	2,1	16,5	21,2	18,3	20,4	17,1	18,5	18,7	1,7
200	26,4	33,5	28,1	31,9	27,2	30,9	29,7	2,6	5,1	9,4	6,5	8,2	5,8	7,0	7,0	1,4

48h

Dose (µmol L^{-1})	Cis_E1	Cis_E2	Cis_E3	Cis_E4	Cis_E5	Cis_E6	Media	S.D.	Pt_E1	Pt_E2	Pt_E3	Pt_E4	Pt_E5	Pt_E6	Media	S.D.
0	100,0	100,0	100,0	100,0	100,0	100,0	100,0	0,0	100,0	100,0	100,0	100,0	100,0	100,0	100,0	0,0
1	89,5	93,4	90,1	92,8	88,9	91,3	91,0	1,7	49,2	55,4	52,4	54,1	50,3	52,6	52,3	2,1
10	49,1	54,3	51,6	53,8	49,9	51,3	51,7	1,9	1,1	4,8	2,1	3,9	1,5	2,6	2,7	1,3
100	14,2	21,3	16,5	19,8	15,2	17,0	17,3	2,5	0,0	0,8	0,2	0,6	0,0	0,4	0,3	0,3
200	12,1	18,4	13,5	16,9	12,8	14,3	14,7	2,3	0,0	0,1	0,0	0,2	0,0	0,1	0,1	0,1

Time dipendenza Viability

Time (h)	Cis_E1	Cis_E2	Cis_E3	Cis_E4	Cis_E5	Cis_E6	Media	S.D.	Pt_E1	Pt_E2	Pt_E3	Pt_E4	Pt_E5	Pt_E6	Media	S.D.
0	100,0	100,0	100,0	100,0	100,0	100,0	100,0	0,0	100,0	100,0	100,0	100,0	100,0	100,0	100,0	0,0
1	98,1	101,4	97,5	100,3	98,4	100,3	99,3	1,4	75,4	83,2	78,4	81,5	76,2	79,3	79,0	2,7
3	96,4	102,1	98,5	101,2	97,3	98,5	99,0	2,0	62,1	69,8	66,1	68,4	63,5	66,1	66,0	2,6
6	90,2	95,4	91,4	94,8	90,9	93,3	92,7	2,0	51,2	57,3	53,5	56,4	52,1	54,5	54,2	2,2
9	81,4	86,2	83,4	85,9	82,1	85,0	84,0	1,8	37,1	44,5	40,4	43,1	38,2	41,7	40,8	2,6
12	53,4	58,1	54,5	57,4	53,9	56,7	55,7	1,8	26,2	33,4	29,4	31,9	27,5	31,0	29,9	2,5
24	29,1	35,4	31,5	34,8	30,2	32,0	32,2	2,3	8,4	13,5	10,1	12,4	9,0	10,6	10,7	1,8
48	11,2	16,4	13,4	15,8	12,1	13,1	13,7	1,9	0,0	1,5	0,5	1,1	0,1	0,8	0,7	0,5

Clonogenic Saggio (n° colonies % of control)

Dose (µmol L^{-1})	Cis_E1	Cis_E2	Cis_E3	Cis_E4	Cis_E5	Cis_E6	Media	S.D.	Pt_E1	Pt_E2	Pt_E3	Pt_E4	Pt_E5	Pt_E6	Media	S.D.
0	100,0	100,0	100,0	100,0	100,0	100,0	100,0	0,0	100,0	100,0	100,0	100,0	100,0	100,0	100,0	0,0
0,1	94,2	99,1	95,7	98,4	94,9	97,7	96,7	1,8	45,1	52,3	47,6	50,9	46,2	49,9	48,7	2,6
0,5	88,4	94,2	90,4	93,1	89,3	92,6	91,3	2,1	38,2	44,9	40,9	43,8	39,1	43,1	41,7	2,5
1	82,1	87,5	84,6	86,9	83,4	85,9	85,1	1,9	28,4	34,7	31,9	33,8	29,5	31,7	31,7	2,2
2,5	52,3	57,4	54,7	56,8	53,1	56,1	55,1	1,9	14,1	20,3	16,6	19,2	15,0	17,8	17,2	2,2
5	47,1	52,8	49,2	51,9	48,7	51,1	50,1	2,0	3,2	7,9	4,9	6,8	3,9	6,3	5,5	1,6

IC50

Gruppo	E1	E2	E3	E4	E5	E6	Media	S.D.
**Cisplatin 24h**	46,25	47,45	46,73	47,51	46,12	46,80	**46,81**	**0,58**
**Ptac2S 24h**	3,82	4,04	3,90	4,01	3,78	3,97	**3,92**	**0,10**
**Cisplatin 48h**	10,88	11,68	11,22	11,75	10,91	11,12	**11,26**	**0,38**
**Ptac2S 48h**	0,83	1,11	1,00	1,15	0,81	0,99	**0,98**	**0,14**

Analisi del Ciclo Cellulare (N=6)

Distribuzione delle Fasi del Ciclo Cellulare (%)

4,0	Fase	E1 (Fig)	E2	E3	E4	E5	E6	Media	S.D.
**Control**	subG1	4,0	3,5	4,5	3,8	4,2	4,0	**4,0**	**0,3405877**
Control	G0/G1	40,6	42,1	39,1	41,5	39,8	40,5	**40,6**	**1,0917875**
Control	S	18,50	17,90	19,10	18,20	18,80	18,50	**18,50**	**0,4242641**
Control	G2/M	36,9	36,5	37,3	36,5	37,2	37,0	**36,9**	**0,3405877**
**Ptac2S**	subG1	77,7	73,2	78,3	75,1	78,6	75,5	**76,4**	**2,1391587**
Ptac2S	G0/G1	20,7	24,1	20,1	22,8	19,9	22,1	**21,6**	**1,6666333**
Ptac2S	S	1,61	2,10	1,30	1,80	1,40	1,81	**1,67**	**0,2948898**
Ptac2S	G2/M	0,0	0,6	0,3	0,3	0,1	0,5	**0,3**	**0,2280351**
**Cisplatin**	subG1	49,9	55,4	51,3	54,1	50,2	52,3	**52,2**	**2,1890637**
Cisplatin	G0/G1	23,0	21,2	24,5	22,1	24,1	22,5	**22,9**	**1,2409674**
Cisplatin	S	25,10	21,40	22,20	21,80	23,70	23,20	**22,90**	**1,3798551**
Cisplatin	G2/M	2,1	2,2	1,9	1,7	2,1	2,4	**2,1**	**0,242212**

Popolazione sub-G1

Gruppo	E1	E2	E3	E4	E5	E6	Media	S.D.
**Control**	4,0	3,5	4,5	3,8	4,2	4,0	**4,0**	**0,34**
**Ptac2S**	77,7	73,2	78,3	75,1	78,6	75,5	**76,4**	**2,14**
**Cisplatin**	49,9	55,4	51,3	54,1	50,2	52,3	**52,2**	**2,19**

ZL55 cells treated with 5 μM Ptac2S (n = 5)

	Time 0h (%)	Time 3h (%)	Time 6h (%)	Time 12h (%)	Time 24h (%)
Rep 1	3,5	32,1	42,3	66,5	76,1
Rep 2	12,1	39,4	51,2	78,4	85,3
Rep 3	5,2	34,8	49,0	73,1	78,9
Rep 4	9,8	41,2	44,1	64,9	83,4
Rep 5	1,4	35,5	45,4	75,1	77,8
**Media**	**6,40**	**36,60**	**46,40**	**71,60**	**80,30**
**Dev. Std**	**4,44**	**3,66**	**3,64**	**5,74**	**3,89**

Cisplatin (CDDP) Apoptosis

ZL55 cells treated with 50 μM CDDP (n = 5)

	Time 0h (%)	Time 3h (%)	Time 6h (%)	Time 12h (%)	Time 24h (%)
Rep 1	2,1	4,2	6,5	44,1	54,3
Rep 2	10,4	11,1	16,2	56,8	63,1
Rep 3	4,8	3,9	11,3	48,3	59,5
Rep 4	7,9	8,5	14,1	54,2	52,8
Rep 5	1,3	5,8	4,9	45,6	58,3
**Media**	**5,30**	**6,70**	**10,60**	**49,80**	**57,60**
**Dev. Std**	**3,85**	**3,06**	**4,83**	**5,49**	**4,13**

Caspasi

	n	Ratio f-Casp-9 / β-actin	Ratio f-Casp-7 / β-actin
Ptac2S - 3 ore	Exp 1	0	0
Ptac2S - 3 ore	Exp 2	0	0
Ptac2S - 3 ore	Exp 3	0	0,1
Ptac2S - 3 ore	Exp 4	0	0
Ptac2S - 3 ore	Exp 5	0	0
Ptac2S - 3 ore	Exp 1	0,41	0,25
Ptac2S - 3 ore	Exp 2	0,48	0,35
Ptac2S - 3 ore	Exp 3	0,39	0,22
Ptac2S - 3 ore	Exp 4	0,51	0,38
Ptac2S - 3 ore	Exp 5	0,46	0,30
Ptac2S - 6 ore	Exp 1	0,98	0,45
Ptac2S - 6 ore	Exp 2	1,15	0,60
Ptac2S - 6 ore	Exp 3	0,92	0,41
Ptac2S - 6 ore	Exp 4	1,18	0,62
Ptac2S - 6 ore	Exp 5	1,07	0,52
Ptac2S - 12 h	Exp 1	1,55	0,70
Ptac2S - 12 h	Exp 2	1,72	0,88
Ptac2S - 12 h	Exp 3	1,51	0,68
Ptac2S - 12 h	Exp 4	1,70	0,86
Ptac2S - 12 h	Exp 5	1,62	0,78
Ptac2S - 24 ore	Exp 1	1,52	0,69
Ptac2S - 24 ore	Exp 2	1,71	0,85
Ptac2S - 24 ore	Exp 3	1,53	0,67
Ptac2S - 24 ore	Exp 4	1,73	0,87
Ptac2S - 24 ore	Exp 5	1,61	0,76

Time	Target Protein	Mean Value	Std Dev (S.D.)	Significance Group	ANOVA P-value
3 ore	f-Casp-9 (	0,45	0,05	a'	< 0.0001
Ptac2S - 3 ore	f-Casp-7	0,3	0,07	A	< 0.0001
6 ore	f-Casp-9	1,06	0,11	b'	< 0.0001
6 ore	f-Casp-7	0,52	0,09	B	< 0.0001
12 ore	f-Casp-9	1,62	0,09	c'	< 0.0001
12 ore	f-Casp-7	0,78	0,09	C	< 0.0001
24 ore	f-Casp-9	1,62	0,1	c'	< 0.0001
24 oe	f-Casp-7	0,77	0,09	C	< 0.0001

Cispatino Time (hours)	Replicate	f-Casp-3 / β-actin	f-Casp-7 / β-actin	f-Casp-9 / β-actin
0	Exp 1	0,00	0,00	0,00
0	Exp 2	0,00	0,00	0,00
0	Exp 3	0,00	0,00	0,00
0	Exp 4	0,00	0,00	0,00
0	Exp 5	0,00	0,00	0,00
3	Exp 1	0,00	0,00	0,00
3	Exp 2	0,00	0,00	0,00
3	Exp 3	0,00	0,00	0,00
3	Exp 4	0,00	0,00	0,00
3	Exp 5	0,00	0,00	0,00
6	Exp 1	0,00	0,16	0,00
6	Exp 2	0,00	0,24	0,00
6	Exp 3	0,00	0,15	0,00
6	Exp 4	0,00	0,26	0,00
6	Exp 5	0,00	0,19	0,00
12	Exp 1	0,28	0,72	0,18
12	Exp 2	0,38	0,86	0,28
12	Exp 3	0,26	0,70	0,16
12	Exp 4	0,39	0,85	0,29
12	Exp 5	0,31	0,77	0,21
24	Exp 1	0,65	0,70	1,41
24	Exp 2	0,79	0,84	1,58
24	Exp 3	0,66	0,68	1,39
24	Exp 4	0,81	0,85	1,61
24	Exp 5	0,71	0,75	1,49

Time	Protein	Media	S.D.	Significatività	ANOVA P-value
0 h		0	0	-	N.D.
3 h		0	0	-	N.D.
6 h	f-Casp-3	0	0	-	N.D.
6 h	f-Casp-7	0,2	0,05	A	< 0.0001
6 h	f-Casp-9	0	0	-	N.D.
12 h	f-Casp-3	0,32	0,06	a	< 0.0001
12 h	f-Casp-7	0,78	0,07	B	< 0.0001
12 h	f-Casp-9	0,22	0,06	a'	< 0.0001
24 h	f-Casp-3	0,72	0,07	b	< 0.0001

꞉AMMENTO PARP

**Ptac2S** - Time (hours)	n	Ratio f-PARP-1 / H3/4
0	Exp 1	0,00
0	Exp 2	0,00
0	Exp 3	0,00
0	Exp 4	0,00
0	Exp 5	0,00
3	Exp 1	0,42
3	Exp 2	0,54
3	Exp 3	0,39
3	Exp 4	0,56
3	Exp 5	0,44
6	Exp 1	0,55
6	Exp 2	0,68
6	Exp 3	0,52
6	Exp 4	0,69
6	Exp 5	0,61
12	Exp 1	0,74
12	Exp 2	0,87
12	Exp 3	0,73
12	Exp 4	0,88
12	Exp 5	0,78
24	Exp 1	0,95
24	Exp 2	1,08
24	Exp 3	0,96
24	Exp 4	1,09
24	Exp 5	1,02

Time		Media	S.D.	Significatività	ANOVA P-value
0 h	f-PARP-1 / H3/4	0,00	0,00	-	N.D.
3 h	f-PARP-1 / H3/4	0,47	0,08	a	< 0.0001
6 h	f-PARP-1 / H3/4	0,61	0,08	ab	< 0.0001
12 h	f-PARP-1 / H3/4	0,80	0,07	b	< 0.0001
24 h	f-PARP-1 / H3/4	1,02	0,07	c	< 0.0001

CISPATINO

Time (hours)	N	Ratio f-PARP-1 / H3/4
0	Exp 1	0,00
0	Exp 2	0,00
0	Exp 3	0,00
0	Exp 4	0,00
0	Exp 5	0,00
3	Exp 1	0,00
3	Exp 2	0,00
3	Exp 3	0,00
3	Exp 4	0,00
3	Exp 5	0,00
6	Exp 1	0,00
6	Exp 2	0,00
6	Exp 3	0,00
6	Exp 4	0,00
6	Exp 5	0,00
12	Exp 1	0,33
12	Exp 2	0,44
12	Exp 3	0,31
12	Exp 4	0,45
12	Exp 5	0,37
24	Exp 1	0,32
24	Exp 2	0,43
24	Exp 3	0,34
24	Exp 4	0,46
24	Exp 5	0,35

Time Point		Media	S.D.	Significatività	ANOVA P-value
0 h	f-PARP-1 / H3/4	0	0	-	N.D.
3 h	f-PARP-1 / H3/4	0	0	-	N.D.
6 h	f-PARP-1 / H3/4	0	0	-	N.D.
12 h	f-PARP-1 / H3/4	0,38	0,06	a	< 0.0001
24 h	f-PARP-1 / H3/4	0,38	0,06	a	< 0.0001

BAX e BCL2 Ptac2S

Time (hours)	n	Ratio Bcl-2 / β-actin	Ratio BAX / β-actin
0,00	Exp 1	0,78	0,44
0,00	Exp 2	0,86	0,55
0,00	Exp 3	0,80	0,42
0,00	Exp 4	0,87	0,56
0,00	Exp 5	0,79	0,53
3,00	Exp 1	0,61	0,65
3,00	Exp 2	0,71	0,79
3,00	Exp 3	0,63	0,68
3,00	Exp 4	0,70	0,81
3,00	Exp 5	0,65	0,72
6,00	Exp 1	0,42	0,92
6,00	Exp 2	0,51	1,08
6,00	Exp 3	0,43	0,94
6,00	Exp 4	0,49	1,07
6,00	Exp 5	0,45	0,99
12,00	Exp 1	0,14	0,91
12,00	Exp 2	0,23	1,06
12,00	Exp 3	0,15	0,93
12,00	Exp 4	0,22	1,08
12,00	Exp 5	0,16	0,92
24,00	Exp 1	0,00	0,98
24,00	Exp 2	0,04	1,12
24,00	Exp 3	0,00	0,99
24,00	Exp 4	0,02	1,11
24,00	Exp 5	0,00	1,05

Time	Proteine	Media	S.D.	Significance Group	ANOVA P-value
0 h	Bcl-2	0,82	0,04	a	< 0.0001
0 h	BAX	0,5	0,07	a'	< 0.001
3 h	Bcl-2	0,66	0,04	b	< 0.0001
3 h	BAX	0,73	0,07	b'	< 0.001
6 h	Bcl-2	0,46	0,04	c	< 0.0001
6 h	BAX	1	0,07	c'	< 0.001
12 h	Bcl-2	0,18	0,04	d	< 0.0001
12 h	BAX	0,98	0,08	c'	< 0.001
24 h	Bcl-2	0,01	0,02	e	< 0.0001
24 h	BAX	1,05	0,07	c'	< 0.001

BAX e BCL2 CISPATINO

Time (hours)	n	Ratio Bcl-2 / β-actin	Ratio BAX / β-actin
0	Exp 1	0,78	0,44
0	Exp 2	0,86	0,55
0	Exp 3	0,80	0,42
0	Exp 4	0,87	0,56
0	Exp 5	0,79	0,53
3	Exp 1	0,61	0,65
3	Exp 2	0,71	0,79
3	Exp 3	0,63	0,68
3	Exp 4	0,70	0,81
3	Exp 5	0,65	0,72
6	Exp 1	0,42	0,92
6	Exp 2	0,51	1,08
6	Exp 3	0,43	0,94
6	Exp 4	0,49	1,07
6	Exp 5	0,45	0,99
12	Exp 1	0,14	0,91
12	Exp 2	0,23	1,06
12	Exp 3	0,15	0,93
12	Exp 4	0,22	1,08
12	Exp 5	0,16	0,92
24	Exp 1	0,00	0,98
24	Exp 2	0,04	1,12
24	Exp 3	0,00	0,99
24	Exp 4	0,02	1,11
24	Exp 5	0,00	1,05

Time	Protein	Media	S.D.	Significatività	ANOVA P-value
0 h	Bcl-2	0,82	0,04	a	< 0.0001
0 h	BAX	0,5	0,07	a'	< 0.001
3 h	Bcl-2	0,66	0,04	b	< 0.0001
3 h	BAX	0,73	0,07	b'	< 0.001
6 h	Bcl-2	0,46	0,04	c	< 0.0001
6 h	BAX	1	0,07	c'	< 0.001
12 h	Bcl-2	0,18	0,04	d	< 0.0001
12 h	BAX	0,98	0,08	c'	< 0.001
24 h	Bcl-2	0,01	0,02	e	< 0.0001
24 h	BAX	1,05	0,07	c'	< 0.001

Time (hours)	n	Control Ratio	Ptac2S Treated Ratio
0	Exp 1	1,44	1,42
0	Exp 2	1,32	1,34
0	Exp 3	1,39	1,45
0	Exp 4	1,41	1,31
0	Exp 5	1,35	1,39
0	Exp 6	1,37	1,37
0,5	Exp 1	1,46	1,14
0,5	Exp 2	1,31	1,05
0,5	Exp 3	1,42	1,08
0,5	Exp 4	1,34	1,16
0,5	Exp 5	1,40	1,04
0,5	Exp 6	1,35	1,13
1	Exp 1	1,42	0,89
1	Exp 2	1,32	0,79
1	Exp 3	1,45	0,84
1	Exp 4	1,31	0,91
1	Exp 5	1,39	0,80
1	Exp 6	1,34	0,87
1,5	Exp 1	1,44	0,64
1,5	Exp 2	1,30	0,52
1,5	Exp 3	1,43	0,58
1,5	Exp 4	1,32	0,67
1,5	Exp 5	1,38	0,55
1,5	Exp 6	1,35	0,64
2	Exp 1	1,43	0,49
2	Exp 2	1,29	0,39
2	Exp 3	1,42	0,44
2	Exp 4	1,31	0,52
2	Exp 5	1,37	0,42
2	Exp 6	1,34	0,50

Time(h)	Guppo	Media	S.D.	Significance (* p < 0.05)
0.0 h	Control	1,38	0,04	No
0.0 h	Ptac2S	1,38	0,05	No
0.5 h	Control	1,38	0,06	No
0.5 h	Ptac2S	1,1	0,05	Yes (*)
1.0 h	Control	1,37	0,06	No
1.0 h	Ptac2S	0,85	0,05	Yes (*)
1.5 h	Control	1,37	0,06	No
1.5 h	Ptac2S	0,6	0,06	Yes (*)
2.0 h	Control	1,36	0,06	No
2.0 h	Ptac2S	0,46	0,05	Yes (*)

Mitocondrio

Time (minuti)	n	Ratio Cit. C / Porin	Ratio BAX / Porin	Ratio Bcl-2 / Porin
0	Exp 1	1,52	0,03	1,91
0	Exp 2	1,71	0,08	2,05
0	Exp 3	1,58	0,04	1,95
0	Exp 4	1,69	0,06	2,04
0	Exp 5	1,59	0,05	1,96
0	Exp 6	1,65	0,04	1,97
15	Exp 1	0,61	1,02	1,93
15	Exp 2	0,74	1,21	2,08
15	Exp 3	0,65	1,07	1,96
15	Exp 4	0,72	1,19	2,07
15	Exp 5	0,66	1,10	2,00
15	Exp 6	0,70	1,14	2,02
30	Exp 1	0,51	1,50	1,56
30	Exp 2	0,62	1,71	1,81
30	Exp 3	0,53	1,54	1,62
30	Exp 4	0,60	1,68	1,77
30	Exp 5	0,55	1,57	1,66
30	Exp 6	0,57	1,61	1,66
60	Exp 1	0,12	1,76	0,98
60	Exp 2	0,21	1,99	1,13
60	Exp 3	0,14	1,83	1,02
60	Exp 4	0,20	1,96	1,12
60	Exp 5	0,16	1,86	1,05
60	Exp 6	0,19	1,88	1,06
120	Exp 1	0,00	1,98	0,70
120	Exp 2	0,05	2,21	0,91
120	Exp 3	0,00	2,04	0,74
120	Exp 4	0,04	2,18	0,88
120	Exp 5	0,01	2,07	0,78
120	Exp 6	0,02	2,12	0,79

Time	Proteine	Mean Value	S.D.	Significatività	ANOVA P-value
0 min	Cit. C	1,62	0,07	a	< 0.0001
0 min	BAX	0,05	0,02	a'	< 0.0001
0 min	Bcl-2	1,98	0,05	A	< 0.0001
15 min	Cit. C	0,68	0,05	b	< 0.0001
15 min	BAX	1,12	0,07	b'	< 0.0001
15 min	Bcl-2	2,01	0,06	B	< 0.0001
30 min	Cit. C	0,56	0,04	c	< 0.0001
30 min	BAX	1,6	0,08	c'	< 0.0001
30 min	Bcl-2	1,68	0,09	B	< 0.0001
60 min	Cit. C	0,17	0,04	d	< 0.0001
60 min	BAX	1,88	0,08	d'	< 0.0001
60 min	Bcl-2	1,06	0,06	C	< 0.0001
120 min	Cit. C	0,02	0,02	e	< 0.0001
120 min	BAX	2,1	0,09	e'	< 0.0001
120 min	Bcl-2	0,8	0,08	D	< 0.0001

CITOPLASMA

Time (minutes)	n	Ratio Cit. C / β-actin	Ratio BAX / β-actin	Ratio Bcl-2 / β-actin
0	Exp 1	0,02	1,85	0,21
0	Exp 2	0,08	2,06	0,29
0	Exp 3	0,04	1,91	0,23
0	Exp 4	0,07	2,02	0,28
0	Exp 5	0,05	1,93	0,24
0	Exp 6	0,04	1,93	0,25
15	Exp 1	0,01	1,61	0,46
15	Exp 2	0,07	1,79	0,58
15	Exp 3	0,03	1,66	0,49
15	Exp 4	0,08	1,76	0,56
15	Exp 5	0,06	1,68	0,51
15	Exp 6	0,05	1,70	0,53
30	Exp 1	0,44	1,29	0,94
30	Exp 2	0,56	1,47	1,07
30	Exp 3	0,47	1,34	0,97
30	Exp 4	0,54	1,44	1,04
30	Exp 5	0,49	1,36	0,99
30	Exp 6	0,50	1,38	1,01
60	Exp 1	0,59	0,81	1,51
60	Exp 2	0,72	0,98	1,73
60	Exp 3	0,61	0,86	1,57
60	Exp 4	0,69	0,95	1,69
60	Exp 5	0,64	0,89	1,60
60	Exp 6	0,67	0,91	1,63
120	Exp 1	1,45	0,00	1,74
120	Exp 2	1,66	0,04	1,96
120	Exp 3	1,49	0,00	1,80
120	Exp 4	1,62	0,03	1,92
120	Exp 5	1,53	0,01	1,83
120	Exp 6	1,56	0,02	1,85

Time	Proteine	Media	S.D.	Significatività	ANOVA P-value
0 min	Cit. C	0,05		a	< 0.0001
0 min	BAX	1,95	0,08	a'	< 0.0001
0 min	Bcl-2	0,25	0,03	A	< 0.0001
15 min	Cit. C	0,05	0,03	a	< 0.0001
15 min	BAX	1,7	0,07	b'	< 0.0001
15 min	Bcl-2	0,52	0,04	B	< 0.0001
30 min	Cit. C	0,5	0,04	b	< 0.0001
30 min	BAX	1,38	0,07	c'	< 0.0001
30 min	Bcl-2	1	0,05	C	< 0.0001
60 min	Cit. C	0,65	0,05	b	< 0.0001
60 min	BAX	0,9	0,06	d'	< 0.0001
60 min	Bcl-2	1,62	0,08	D	< 0.0001
120 min	Cit. C	1,55	0,08	c	< 0.0001
120 min	BAX	0,02	0,02	e'	< 0.0001
120 min	Bcl-2	1,85	0,08	D	< 0.0001

Figura 4

B

Time (hours)	n	Ratio f-PKCδ / Porin
0	Exp 1	0,00
0	Exp 2	0,00
0	Exp 3	0,00
0	Exp 4	0,00
0	Exp 5	0,00
0	Exp 6	0,00
0,5	Exp 1	0,79
0,5	Exp 2	0,97
0,5	Exp 3	0,84
0,5	Exp 4	0,94
0,5	Exp 5	0,87
0,5	Exp 6	0,87
1	Exp 1	1,03
1	Exp 2	1,20
1	Exp 3	1,07
1	Exp 4	1,16
1	Exp 5	1,09
1	Exp 6	1,11
3	Exp 1	1,10
3	Exp 2	1,33
3	Exp 3	1,16
3	Exp 4	1,28
3	Exp 5	1,19
3	Exp 6	1,21
6	Exp 1	1,50
6	Exp 2	1,78
6	Exp 3	1,58
6	Exp 4	1,71
6	Exp 5	1,62
6	Exp 6	1,65
12	Exp 1	1,40
12	Exp 2	1,74
12	Exp 3	1,49
12	Exp 4	1,67
12	Exp 5	1,53
12	Exp 6	1,52

	Proteina	Time	Media	S.D.	Significatività
Ptac2S (5 μM)	f-PKCδ	0 h	0,00	0,00	-
Ptac2S (5 μM)	f-PKCδ	0.5 h	0,88	0,06	a
Ptac2S (5 μM)	f-PKCδ	1 h	1,11	0,06	bc
Ptac2S (5 μM)	f-PKCδ	3 h	1,21	0,08	c
Ptac2S (5 μM)	f-PKCδ	6 h	1,64	0,09	d
Ptac2S (5 μM)	f-PKCδ	12 h	1,55	0,11	d

C

Tempo (min)	n	PKCε Citoplasma (β-actin)	PKCε Membrana (Na+/K+ ATPase)	PKCε Nucleo (H3/4)
0	Exp 1	1,14	0,00	0,01
0	Exp 2	1,25	0,00	0,04
0	Exp 3	1,18	0,00	0,02
0	Exp 4	1,22	0,00	0,01
0	Exp 5	1,19	0,00	0,03
0	Exp 6	1,22	0,00	0,01
2,5	Exp 1	0,63	0,84	0,01
2,5	Exp 2	0,74	0,96	0,05
2,5	Exp 3	0,66	0,88	0,03
2,5	Exp 4	0,71	0,93	0,02
2,5	Exp 5	0,67	0,89	0,04
2,5	Exp 6	0,67	0,90	0,03
5	Exp 1	0,38	0,94	0,02
5	Exp 2	0,47	1,06	0,05
5	Exp 3	0,40	0,97	0,02
5	Exp 4	0,45	1,03	0,01
5	Exp 5	0,41	0,99	0,04
5	Exp 6	0,41	1,01	0,04
10	Exp 1	0,23	1,23	0,01
10	Exp 2	0,34	1,37	0,06
10	Exp 3	0,26	1,27	0,03
10	Exp 4	0,31	1,34	0,02
10	Exp 5	0,27	1,29	0,04
10	Exp 6	0,27	1,30	0,02
20	Exp 1	0,00	1,14	0,01
20	Exp 2	0,00	1,26	0,04
20	Exp 3	0,00	1,17	0,02
20	Exp 4	0,00	1,23	0,01
20	Exp 5	0,00	1,19	0,03
20	Exp 6	0,00	1,21	0,01

Compartimento Subcellulare	Tempo (min)	Riferimento di Carico	Media	Deviazione Standard (S.D.)	Gruppo Significatività (Bonferroni/Dunn)
Citoplasma	0 min	β-actin	1,2	0,04	a
Citoplasma	2.5 min	β-actin	0,68	0,04	b
Citoplasma	5 min	β-actin	0,42	0,03	c
Citoplasma	10 min	β-actin	0,28	0,04	d
Citoplasma	20 min	β-actin	0	0	-
Membrana	0 min	α-subunit of Na+/K+	0	0	-
Membrana	2.5 min	α-subunit of Na+/K+	0,9	0,04	a'
Membrana	5 min	α-subunit of Na+/K+	1	0,04	b'
Membrana	10 min	α-subunit of Na+/K+	1,3	0,05	c'
Membrana	20 min	α-subunit of Na+/K+	1,2	0,04	c'
Nucleo	0 min	H3/4	0,02	0,01	A
Nucleo	2.5 min	H3/4	0,03	0,01	A
Nucleo	5 min	H3/4	0,03	0,02	A
Nucleo	10 min	H3/4	0,03	0,02	A
Nucleo	20 min	H3/4	0,02	0,01	A

D

Tempo (min)	Replica	PKCδ cyt (β-actin)	PKCδ mem (Na+/K+ ATPase)	PKCδ nuc (H3/4)
0	Exp 1	0,98	0,49	0,02
0	Exp 2	1,10	0,58	0,06
0	Exp 3	1,02	0,52	0,03
0	Exp 4	1,06	0,55	0,05
0	Exp 5	1,01	0,53	0,02
0	Exp 6	1,07	0,57	0,06
2,5	Exp 1	0,80	1,32	0,39
2,5	Exp 2	0,91	1,48	0,51
2,5	Exp 3	0,84	1,37	0,43
2,5	Exp 4	0,89	1,43	0,47
2,5	Exp 5	0,83	1,38	0,42
2,5	Exp 6	0,89	1,44	0,48
5	Exp 1	0,64	1,30	1,12
5	Exp 2	0,74	1,45	1,27
5	Exp 3	0,68	1,35	1,16
5	Exp 4	0,72	1,41	1,23
5	Exp 5	0,67	1,36	1,18
5	Exp 6	0,75	1,41	1,24
10	Exp 1	0,11	1,74	1,36
10	Exp 2	0,21	1,89	1,51
10	Exp 3	0,14	1,79	1,41
10	Exp 4	0,18	1,85	1,48
10	Exp 5	0,13	1,80	1,41
10	Exp 6	0,19	1,85	1,48
20	Exp 1	0,00	1,81	1,51
20	Exp 2	0,00	1,98	1,69
20	Exp 3	0,00	1,86	1,56
20	Exp 4	0,00	1,93	1,64
20	Exp 5	0,00	1,87	1,58
20	Exp 6	0,00	1,95	1,62

Compartimento Subcellulare	Tempo (min)	Controllo di Carico	Media	Deviazione Standard (S.D.)	Gruppo Significatività (Bonferroni/Dunn)
Citoplasma (cyt)	0 min	β-actin	1,04	0,04	a
Citoplasma (cyt)	2.5 min	β-actin	0,86	0,04	b
Citoplasma (cyt)	5 min	β-actin	0,7	0,04	c
Citoplasma (cyt)	10 min	β-actin	0,16	0,04	d
Citoplasma (cyt)	20 min	β-actin	0	0	-
Membrana (mem)	0 min	α-subunit of Na+/K+	0,54	0,03	a'
Membrana (mem)	2.5 min	α-subunit of Na+/K+	1,4	0,06	b'
Membrana (mem)	5 min	α-subunit of Na+/K+	1,38	0,05	b'
Membrana (mem)	10 min	α-subunit of Na+/K+	1,82	0,05	c'
Membrana (mem)	20 min	α-subunit of Na+/K+	1,9	0,06	c'
Nucleo (nuc)	0 min	H3/4	0,04	0,02	A
Nucleo (nuc)	2.5 min	H3/4	0,45	0,04	B
Nucleo (nuc)	5 min	H3/4	1,2	0,06	C
Nucleo (nuc)	10 min	H3/4	1,44	0,06	D
Nucleo (nuc)	20 min	H3/4	1,6	0,06	D

E

Tempo (h)	Condizione Sperimentale	JC-1 Fluorescence Ratio (Red/Green)
0	Controllo	1,41
0	Controllo	1,48
0	Controllo	1,35
0	Controllo	1,43
0	Controllo	1,37
0	Controllo	1,36
0	Ptac2S	1,41
0	Ptac2S	1,48
0	Ptac2S	1,35
0	Ptac2S	1,43
0	Ptac2S	1,37
0	Ptac2S	1,36
0	siRNA-PKC-δ +	1,41
0	siRNA-PKC-δ +	1,48
0	siRNA-PKC-δ +	1,35
0	siRNA-PKC-δ +	1,43
0	siRNA-PKC-δ +	1,37
0	siRNA-PKC-δ +	1,36
0,5	Controllo	1,38
0,5	Controllo	1,49
0,5	Controllo	1,34
0,5	Controllo	1,44
0,5	Controllo	1,39
0,5	Controllo	1,36
0,5	Ptac2S	1,05
0,5	Ptac2S	1,16
0,5	Ptac2S	1,08
0,5	Ptac2S	1,13
0,5	Ptac2S	1,06
0,5	Ptac2S	1,12
0,5	siRNA-PKC-δ +	1,28
0,5	siRNA-PKC-δ +	1,41
0,5	siRNA-PKC-δ +	1,30
0,5	siRNA-PKC-δ +	1,36
0,5	siRNA-PKC-δ +	1,32
0,5	siRNA-PKC-δ +	1,31
1	Controllo	1,37
1	Controllo	1,49
1	Controllo	1,33
1	Controllo	1,46
1	Controllo	1,38
1	Controllo	1,37
1	Ptac2S	0,79
1	Ptac2S	0,91
1	Ptac2S	0,82
1	Ptac2S	0,88
1	Ptac2S	0,81
1	Ptac2S	0,89
1	siRNA-PKC-δ +	1,24
1	siRNA-PKC-δ +	1,37
1	siRNA-PKC-δ +	1,27
1	siRNA-PKC-δ +	1,33
1	siRNA-PKC-δ +	1,29
1	siRNA-PKC-δ +	1,30
1,5	Controllo	1,36
1,5	Controllo	1,48
1,5	Controllo	1,34
1,5	Controllo	1,44
1,5	Controllo	1,39
1,5	Controllo	1,39
1,5	Ptac2S	0,54
1,5	Ptac2S	0,67
1,5	Ptac2S	0,57
1,5	Ptac2S	0,64
1,5	Ptac2S	0,56
1,5	Ptac2S	0,62
1,5	siRNA-PKC-δ +	1,17
1,5	siRNA-PKC-δ +	1,31
1,5	siRNA-PKC-δ +	1,20
1,5	siRNA-PKC-δ +	1,26
1,5	siRNA-PKC-δ +	1,22
1,5	siRNA-PKC-δ +	1,23
2	Controllo	1,34
2	Controllo	1,47
2	Controllo	1,32
2	Controllo	1,43
2	Controllo	1,37
2	Controllo	1,35
2	Ptac2S	0,39
2	Ptac2S	0,52
2	Ptac2S	0,42
2	Ptac2S	0,49
2	Ptac2S	0,41
2	Ptac2S	0,47
2	siRNA-PKC-δ +	1,13
2	siRNA-PKC-δ +	1,28
2	siRNA-PKC-δ +	1,16
2	siRNA-PKC-δ +	1,23
2	siRNA-PKC-δ +	1,19
2	siRNA-PKC-δ +	1,21

Tempo (h)	Condizione Sperimentale	Media (Mean)	Deviazione Standard (S.D.)	Significatività vs Controllo
0 h	Controllo	1,4	0,05	n.s.
0 h	Ptac2S	1,4	0,05	n.s.
0 h	siRNA-PKC-δ +	1,4	0,05	n.s.
0.5 h	Controllo	1,4	0,06	
0.5 h	Ptac2S	1,1	0,04	* ($p < 0.05$)
0.5 h	siRNA-PKC-δ +	1,33	0,05	n.s.
1 h	Controllo	1,4	0,06	
1 h	Ptac2S	0,85	0,05	* ($p < 0.05$)
1 h	siRNA-PKC-δ +	1,3	0,05	n.s.
1.5 h	Controllo	1,4	0,05	
1.5 h	Ptac2S	0,6	0,05	* ($p < 0.05$)
1.5 h	siRNA-PKC-δ +	1,23	0,05	n.s.
2 h	Controllo	1,38	0,06	
2 h	Ptac2S	0,45	0,05	* ($p < 0.05$)
2 h	siRNA-PKC-δ +	1,2	0,05	n.s.

VITALITA'

Esperimento	ondizione siRN	alità (% del Controllo)
Exp 1	Controllo (-)	96,9
Exp 1	Controllo (-)	98,9
Exp 1	Controllo (-)	98,5
Exp 1	Controllo (-)	102,6
Exp 1	Controllo (-)	102,1
Exp 1	Controllo (-)	103,8
Exp 1	Controllo (-)	97,4
Exp 1	Controllo (-)	100,1
Exp 1	Controllo (-)	43,4
Exp 1	Controllo (-)	45,7
Exp 1	Controllo (-)	41,9
Exp 1	Controllo (-)	43,2
Exp 1	Controllo (-)	46,9
Exp 1	Controllo (-)	46,0
Exp 1	Controllo (-)	43,4

	Condizione siRN	Ptac2S	Dettaglio / Target	Media (Mean)	Deviazione Standard (S.D.)	Gruppo Post-Hoc
Vitalità Cellulare (SRB)	Controllo (-)	No	Vitalità totale	69,9	22,43	a
Vitalità Cellulare (SRB)	Controllo (-)	Sì	Vitalità totale	71,51	23,1	b
Vitalità Cellulare (SRB)	siRNA-NS (Scrar	Sì	Vitalità totale	69,87	22,74	b
Vitalità Cellulare (SRB)	siRNA-PKC-δ	Sì	Vitalità totale	70,18	22,91	c

Exp 1	Controllo (-)	46,4
Exp 1	siRNA-NS (Scra	48,6
Exp 1	siRNA-NS (Scra	55,0
Exp 1	siRNA-NS (Scra	54,1
Exp 1	siRNA-NS (Scra	51,3
Exp 1	siRNA-NS (Scra	49,8
Exp 1	siRNA-NS (Scra	56,2
Exp 1	siRNA-NS (Scra	51,2
Exp 1	siRNA-NS (Scra	49,3
Exp 1	siRNA-PKC-δ	83,8
Exp 1	siRNA-PKC-δ	81,8
Exp 1	siRNA-PKC-δ	83,4
Exp 1	siRNA-PKC-δ	82,8
Exp 1	siRNA-PKC-δ	81,3
Exp 1	siRNA-PKC-δ	84,8
Exp 1	siRNA-PKC-δ	80,0
Exp 1	siRNA-PKC-δ	81,4
Exp 2	Controllo (-)	102,6
Exp 2	Controllo (-)	104,5
Exp 2	Controllo (-)	102,3
Exp 2	Controllo (-)	103,3
Exp 2	Controllo (-)	98,0
Exp 2	Controllo (-)	99,5
Exp 2	Controllo (-)	100,0
Exp 2	Controllo (-)	98,3
Exp 2	Controllo (-)	43,5
Exp 2	Controllo (-)	44,9
Exp 2	Controllo (-)	47,8
Exp 2	Controllo (-)	45,6
Exp 2	Controllo (-)	45,6
Exp 2	Controllo (-)	44,3
Exp 2	Controllo (-)	44,8
Exp 2	Controllo (-)	50,2
Exp 2	siRNA-NS (Scra	52,6
Exp 2	siRNA-NS (Scra	49,1
Exp 2	siRNA-NS (Scra	53,6
Exp 2	siRNA-NS (Scra	49,1
Exp 2	siRNA-NS (Scra	50,8
Exp 2	siRNA-NS (Scra	55,7
Exp 2	siRNA-NS (Scra	52,9
Exp 2	siRNA-NS (Scra	52,2
Exp 2	siRNA-PKC-δ	86,7
Exp 2	siRNA-PKC-δ	86,1
Exp 2	siRNA-PKC-δ	81,8
Exp 2	siRNA-PKC-δ	80,2
Exp 2	siRNA-PKC-δ	82,5
Exp 2	siRNA-PKC-δ	82,1
Exp 2	siRNA-PKC-δ	81,6
Exp 2	siRNA-PKC-δ	87,5
Exp 3	Controllo (-)	100,4
Exp 3	Controllo (-)	103,1
Exp 3	Controllo (-)	101,1
Exp 3	Controllo (-)	105,2
Exp 3	Controllo (-)	101,6
Exp 3	Controllo (-)	100,0
Exp 3	Controllo (-)	99,9
Exp 3	Controllo (-)	102,4
Exp 3	Controllo (-)	47,5
Exp 3	Controllo (-)	50,0
Exp 3	Controllo (-)	46,0
Exp 3	Controllo (-)	44,6
Exp 3	Controllo (-)	50,8
Exp 3	Controllo (-)	46,9
Exp 3	Controllo (-)	43,5
Exp 3	Controllo (-)	43,2
Exp 3	siRNA-NS (Scra	50,1
Exp 3	siRNA-NS (Scra	51,4
Exp 3	siRNA-NS (Scra	48,4
Exp 3	siRNA-NS (Scra	45,6
Exp 3	siRNA-NS (Scra	48,1
Exp 3	siRNA-NS (Scra	53,0
Exp 3	siRNA-NS (Scra	49,3
Exp 3	siRNA-NS (Scra	52,8
Exp 3	siRNA-PKC-δ	80,9
Exp 3	siRNA-PKC-δ	86,6
Exp 3	siRNA-PKC-δ	86,3
Exp 3	siRNA-PKC-δ	85,1
Exp 3	siRNA-PKC-δ	82,9
Exp 3	siRNA-PKC-δ	85,9
Exp 3	siRNA-PKC-δ	81,7
Exp 3	siRNA-PKC-δ	84,3
Exp 4	Controllo (-)	103,4
Exp 4	Controllo (-)	102,8
Exp 4	Controllo (-)	97,9
Exp 4	Controllo (-)	99,8
Exp 4	Controllo (-)	97,2
Exp 4	Controllo (-)	103,1
Exp 4	Controllo (-)	102,7
Exp 4	Controllo (-)	98,2
Exp 4	Controllo (-)	49,6
Exp 4	Controllo (-)	45,9
Exp 4	Controllo (-)	50,8
Exp 4	Controllo (-)	49,0
Exp 4	Controllo (-)	50,9
Exp 4	Controllo (-)	48,9
Exp 4	Controllo (-)	44,7
Exp 4	Controllo (-)	47,3
Exp 4	siRNA-NS (Scra	52,0
Exp 4	siRNA-NS (Scra	51,6
Exp 4	siRNA-NS (Scra	51,3
Exp 4	siRNA-NS (Scra	47,1
Exp 4	siRNA-NS (Scra	45,1
Exp 4	siRNA-NS (Scra	51,6
Exp 4	siRNA-NS (Scra	52,2
Exp 4	siRNA-NS (Scra	45,3
Exp 4	siRNA-PKC-δ	79,5
Exp 4	siRNA-PKC-δ	85,0
Exp 4	siRNA-PKC-δ	85,1
Exp 4	siRNA-PKC-δ	80,0
Exp 4	siRNA-PKC-δ	82,7
Exp 4	siRNA-PKC-δ	83,3
Exp 4	siRNA-PKC-δ	81,1
Exp 4	siRNA-PKC-δ	85,9
Exp 5	Controllo (-)	97,3
Exp 5	Controllo (-)	99,9
Exp 5	Controllo (-)	101,5
Exp 5	Controllo (-)	97,2
Exp 5	Controllo (-)	98,1
Exp 5	Controllo (-)	103,6
Exp 5	Controllo (-)	100,8
Exp 5	Controllo (-)	99,1
Exp 5	Controllo (-)	45,1
Exp 5	Controllo (-)	45,9
Exp 5	Controllo (-)	46,8
Exp 5	Controllo (-)	48,8
Exp 5	Controllo (-)	45,9
Exp 5	Controllo (-)	45,9
Exp 5	Controllo (-)	44,7
Exp 5	Controllo (-)	49,1
Exp 5	siRNA-NS (Scra	52,9
Exp 5	siRNA-NS (Scra	52,5
Exp 5	siRNA-NS (Scra	46,2
Exp 5	siRNA-NS (Scra	47,6
Exp 5	siRNA-NS (Scra	51,0
Exp 5	siRNA-NS (Scra	47,4
Exp 5	siRNA-NS (Scra	46,7
Exp 5	siRNA-NS (Scra	53,1
Exp 5	siRNA-PKC-δ	83,1
Exp 5	siRNA-PKC-δ	85,6
Exp 5	siRNA-PKC-δ	85,8
Exp 5	siRNA-PKC-δ	80,9
Exp 5	siRNA-PKC-δ	80,1
Exp 5	siRNA-PKC-δ	82,8
Exp 5	siRNA-PKC-δ	82,7
Exp 5	siRNA-PKC-δ	83,1
Exp 6	Controllo (-)	102,5
Exp 6	Controllo (-)	105,0
Exp 6	Controllo (-)	97,9
Exp 6	Controllo (-)	100,4
Exp 6	Controllo (-)	99,9
Exp 6	Controllo (-)	104,0
Exp 6	Controllo (-)	99,1
Exp 6	Controllo (-)	98,7
Exp 6	Controllo (-)	47,1
Exp 6	Controllo (-)	46,0
Exp 6	Controllo (-)	45,7
Exp 6	Controllo (-)	51,1
Exp 6	Controllo (-)	47,3
Exp 6	Controllo (-)	50,6
Exp 6	Controllo (-)	48,2
Exp 6	Controllo (-)	44,2
Exp 6	siRNA-NS (Scra	56,2
Exp 6	siRNA-NS (Scra	57,3
Exp 6	siRNA-NS (Scra	56,9
Exp 6	siRNA-NS (Scra	56,3
Exp 6	siRNA-NS (Scra	50,8
Exp 6	siRNA-NS (Scra	53,4
Exp 6	siRNA-NS (Scra	51,2
Exp 6	siRNA-NS (Scra	52,7
Exp 6	siRNA-PKC-δ	79,8
Exp 6	siRNA-PKC-δ	84,7
Exp 6	siRNA-PKC-δ	78,9
Exp 6	siRNA-PKC-δ	83,1
Exp 6	siRNA-PKC-δ	80,4
Exp 6	siRNA-PKC-δ	80,2
Exp 6	siRNA-PKC-δ	84,5
Exp 6	siRNA-PKC-δ	84,8

Esperimento	ondizione siRN	Ptac2S	Proteina	Rapporto di Espressione / β-actin
Exp 1	Controllo (-)	No	f-Casp-7	0,00
Exp 1	Controllo (-)	No	f-PARP-1	0,00
Exp 1	Controllo (-)	Sì	f-Casp-7	0,41
Exp 1	Controllo (-)	Sì	f-PARP-1	0,55
Exp 1	siRNA-NS (Scra	Sì	f-Casp-7	0,47
Exp 1	siRNA-NS (Scra	Sì	f-PARP-1	0,44
Exp 1	siRNA-PKC-δ	Sì	f-Casp-7	0,24
Exp 1	siRNA-PKC-δ	Sì	f-PARP-1	0,22
Exp 2	Controllo (-)	No	f-Casp-7	0,00
Exp 2	Controllo (-)	No	f-PARP-1	0,00
Exp 2	Controllo (-)	Sì	f-Casp-7	0,42
Exp 2	Controllo (-)	Sì	f-PARP-1	0,57
Exp 2	siRNA-NS (Scra	Sì	f-Casp-7	0,50
Exp 2	siRNA-NS (Scra	Sì	f-PARP-1	0,55
Exp 2	siRNA-PKC-δ	Sì	f-Casp-7	0,23
Exp 2	siRNA-PKC-δ	Sì	f-PARP-1	0,27
Exp 3	Controllo (-)	No	f-Casp-7	0,00
Exp 3	Controllo (-)	No	f-PARP-1	0,00
Exp 3	Controllo (-)	Sì	f-Casp-7	0,33
Exp 3	Controllo (-)	Sì	f-PARP-1	0,56
Exp 3	siRNA-NS (Scra	Sì	f-Casp-7	0,48
Exp 3	siRNA-NS (Scra	Sì	f-PARP-1	0,47
Exp 3	siRNA-PKC-δ	Sì	f-Casp-7	0,20
Exp 3	siRNA-PKC-δ	Sì	f-PARP-1	0,21
Exp 4	Controllo (-)	No	f-Casp-7	0,00
Exp 4	Controllo (-)	No	f-PARP-1	0,00
Exp 4	Controllo (-)	Sì	f-Casp-7	0,39
Exp 4	Controllo (-)	Sì	f-PARP-1	0,75
Exp 4	siRNA-NS (Scra	Sì	f-Casp-7	0,49
Exp 4	siRNA-NS (Scra	Sì	f-PARP-1	0,46
Exp 4	siRNA-PKC-δ	Sì	f-Casp-7	0,21
Exp 4	siRNA-PKC-δ	Sì	f-PARP-1	0,27
Exp 5	Controllo (-)	No	f-Casp-7	0,00
Exp 5	Controllo (-)	No	f-PARP-1	0,00
Exp 5	Controllo (-)	Sì	f-Casp-7	0,41
Exp 5	Controllo (-)	Sì	f-PARP-1	0,60
Exp 5	siRNA-NS (Scra	Sì	f-Casp-7	0,45
Exp 5	siRNA-NS (Scra	Sì	f-PARP-1	0,55
Exp 5	siRNA-PKC-δ	Sì	f-Casp-7	0,27
Exp 5	siRNA-PKC-δ	Sì	f-PARP-1	0,25

Ambito Analisi	Condizione siRNA	Ptac2S	proteine	Media (Mean)	Deviazione Standard (S.D.)	Gruppo Post-Hoc
Densitometria WB	Controllo (-)	No	f-Casp-7 / β-actin			**-**
Densitometria WB	Controllo (-)	No	f-PARP-1 /	0	0	**-**
Densitometria WB	Controllo (-)	Sì	f-Casp-7 /	0,39	0,04	**a**
Densitometria WB	Controllo (-)	Sì	f-PARP-1 /	0,6	0,08	**a'**
Densitometria WB	siRNA-NS (Sc	Sì	f-Casp-7 /	0,48	0,02	**a**
Densitometria WB	siRNA-NS (Sc	Sì	f-PARP-1 /	0,49	0,05	**b'**
Densitometria WB	siRNA-PKC-δ	Sì	f-Casp-7 /	0,23	0,03	**b**
Densitometria WB	siRNA-PKC-δ	Sì	f-PARP-1 /	0,24	0,03	**c'**

FIG 5 A

Trattamento	Tempo (h)	Esperimento	Rapporto p-p38MAPK / p38MAPK
Ptac2S (5 µM)	0	Exp 1	0,38
Ptac2S (5 µM)	0	Exp 2	0,61
Ptac2S (5 µM)	0	Exp 3	0,43
Ptac2S (5 µM)	0	Exp 4	0,71
Ptac2S (5 µM)	0	Exp 5	0,60
Ptac2S (5 µM)	1	Exp 1	1,39
Ptac2S (5 µM)	1	Exp 2	1,46
Ptac2S (5 µM)	1	Exp 3	1,37
Ptac2S (5 µM)	1	Exp 4	1,26
Ptac2S (5 µM)	1	Exp 5	1,28
Ptac2S (5 µM)	3	Exp 1	2,69
Ptac2S (5 µM)	3	Exp 2	2,61
Ptac2S (5 µM)	3	Exp 3	2,28
Ptac2S (5 µM)	3	Exp 4	2,55
Ptac2S (5 µM)	3	Exp 5	2,48
Ptac2S (5 µM)	6	Exp 1	2,39
Ptac2S (5 µM)	6	Exp 2	2,81
Ptac2S (5 µM)	6	Exp 3	2,69
Ptac2S (5 µM)	6	Exp 4	2,68
Ptac2S (5 µM)	6	Exp 5	2,80
Ptac2S (5 µM)	12	Exp 1	1,64
Ptac2S (5 µM)	12	Exp 2	1,66
Ptac2S (5 µM)	12	Exp 3	1,99
Ptac2S (5 µM)	12	Exp 4	1,92
Ptac2S (5 µM)	12	Exp 5	2,04
Ptac2S (5 µM)	24	Exp 1	1,13
Ptac2S (5 µM)	24	Exp 2	1,03
Ptac2S (5 µM)	24	Exp 3	1,20
Ptac2S (5 µM)	24	Exp 4	1,17
Ptac2S (5 µM)	24	Exp 5	1,15
Cisplatino (50 µM)	0	Exp 1	0,62
Cisplatino (50 µM)	0	Exp 2	0,60
Cisplatino (50 µM)	0	Exp 3	0,48
Cisplatino (50 µM)	0	Exp 4	0,47
Cisplatino (50 µM)	0	Exp 5	0,57
Cisplatino (50 µM)	1	Exp 1	0,45
Cisplatino (50 µM)	1	Exp 2	0,52
Cisplatino (50 µM)	1	Exp 3	0,74
Cisplatino (50 µM)	1	Exp 4	0,48
Cisplatino (50 µM)	1	Exp 5	0,45
Cisplatino (50 µM)	3	Exp 1	0,82
Cisplatino (50 µM)	3	Exp 2	0,86
Cisplatino (50 µM)	3	Exp 3	0,96
Cisplatino (50 µM)	3	Exp 4	0,99
Cisplatino (50 µM)	3	Exp 5	0,80
Cisplatino (50 µM)	6	Exp 1	1,13
Cisplatino (50 µM)	6	Exp 2	1,07
Cisplatino (50 µM)	6	Exp 3	0,72
Cisplatino (50 µM)	6	Exp 4	0,98
Cisplatino (50 µM)	6	Exp 5	1,10
Cisplatino (50 µM)	12	Exp 1	0,57
Cisplatino (50 µM)	12	Exp 2	0,50
Cisplatino (50 µM)	12	Exp 3	0,41
Cisplatino (50 µM)	12	Exp 4	0,55
Cisplatino (50 µM)	12	Exp 5	0,41
Cisplatino (50 µM)	24	Exp 1	0,45
Cisplatino (50 µM)	24	Exp 2	0,44
Cisplatino (50 µM)	24	Exp 3	0,22
Cisplatino (50 µM)	24	Exp 4	0,49
Cisplatino (50 µM)	24	Exp 5	0,57

Trattamento	Tempo (h)	Media	Dev. Std. (S.D.)	Significatività (Post-Hoc)
Ptac2S (5 µM)	0	0,55	0,14	a
Ptac2S (5 µM)	1	1,35	0,08	b
Ptac2S (5 µM)	3	2,52	0,16	c
Ptac2S (5 µM)	6	2,67	0,17	c
Ptac2S (5 µM)	12	1,85	0,19	d
Ptac2S (5 µM)	24	1,14	0,06	b
Cisplatino (50 µM)	0	0,55	0,07	a
Cisplatino (50 µM)	1	0,53	0,12	a
Cisplatino (50 µM)	3	0,89	0,08	ab
Cisplatino (50 µM)	6	1,00	0,17	b
Cisplatino (50 µM)	12	0,49	0,08	a
Cisplatino (50 µM)	24	0,43	0,13	a

FIG 5D

Condizione Sperimentale	Ptac2S (5 µM)	Esperimento	Replica Tecnica	Vitalità Cellulare (% del Ctrl)
Controllo non trattato (Ctrl N	No	Exp 1	Rep 1	102,5
Controllo non trattato (Ctrl N	No	Exp 1	Rep 2	106,5
Controllo non trattato (Ctrl N	No	Exp 1	Rep 3	102,8
Controllo non trattato (Ctrl N	No	Exp 1	Rep 4	99,0
Controllo non trattato (Ctrl N	No	Exp 1	Rep 5	99,4
Controllo non trattato (Ctrl N	No	Exp 1	Rep 6	98,8
Controllo non trattato (Ctrl N	No	Exp 1	Rep 7	100,7
Controllo non trattato (Ctrl N	No	Exp 1	Rep 8	102,4
Controllo non trattato (Ctrl N	No	Exp 2	Rep 1	102,3
Controllo non trattato (Ctrl N	No	Exp 2	Rep 2	107,3
Controllo non trattato (Ctrl N	No	Exp 2	Rep 3	102,2
Controllo non trattato (Ctrl N	No	Exp 2	Rep 4	101,4
Controllo non trattato (Ctrl N	No	Exp 2	Rep 5	104,4
Controllo non trattato (Ctrl N	No	Exp 2	Rep 6	103,0
Controllo non trattato (Ctrl N	No	Exp 2	Rep 7	98,8
Controllo non trattato (Ctrl N	No	Exp 2	Rep 8	102,8
Controllo non trattato (Ctrl N	No	Exp 3	Rep 1	97,7
Controllo non trattato (Ctrl N	No	Exp 3	Rep 2	97,4
Controllo non trattato (Ctrl N	No	Exp 3	Rep 3	100,1
Controllo non trattato (Ctrl N	No	Exp 3	Rep 4	102,0
Controllo non trattato (Ctrl N	No	Exp 3	Rep 5	99,0
Controllo non trattato (Ctrl N	No	Exp 3	Rep 6	100,5
Controllo non trattato (Ctrl N	No	Exp 3	Rep 7	101,1
Controllo non trattato (Ctrl N	No	Exp 3	Rep 8	95,9
Controllo non trattato (Ctrl N	No	Exp 4	Rep 1	102,5
Controllo non trattato (Ctrl N	No	Exp 4	Rep 2	98,9
Controllo non trattato (Ctrl N	No	Exp 4	Rep 3	98,6
Controllo non trattato (Ctrl N	No	Exp 4	Rep 4	102,4
Controllo non trattato (Ctrl N	No	Exp 4	Rep 5	97,5
Controllo non trattato (Ctrl N	No	Exp 4	Rep 6	102,7
Controllo non trattato (Ctrl N	No	Exp 4	Rep 7	103,5
Controllo non trattato (Ctrl N	No	Exp 4	Rep 8	100,7
Controllo non trattato (Ctrl N	No	Exp 5	Rep 1	99,8
Controllo non trattato (Ctrl N	No	Exp 5	Rep 2	100,6
Controllo non trattato (Ctrl N	No	Exp 5	Rep 3	100,0
Controllo non trattato (Ctrl N	No	Exp 5	Rep 4	99,8
Controllo non trattato (Ctrl N	No	Exp 5	Rep 5	98,8
Controllo non trattato (Ctrl N	No	Exp 5	Rep 6	99,5
Controllo non trattato (Ctrl N	No	Exp 5	Rep 7	101,7
Controllo non trattato (Ctrl N	No	Exp 5	Rep 8	100,5
siRNA Scramble + Ptac2S	Sì	Exp 1	Rep 1	46,6
siRNA Scramble + Ptac2S	Sì	Exp 1	Rep 2	47,4
siRNA Scramble + Ptac2S	Sì	Exp 1	Rep 3	44,3
siRNA Scramble + Ptac2S	Sì	Exp 1	Rep 4	42,5
siRNA Scramble + Ptac2S	Sì	Exp 1	Rep 5	43,2
siRNA Scramble + Ptac2S	Sì	Exp 1	Rep 6	42,6
siRNA Scramble + Ptac2S	Sì	Exp 1	Rep 7	40,1
siRNA Scramble + Ptac2S	Sì	Exp 1	Rep 8	44,7
siRNA Scramble + Ptac2S	Sì	Exp 2	Rep 1	47,9
siRNA Scramble + Ptac2S	Sì	Exp 2	Rep 2	43,0
siRNA Scramble + Ptac2S	Sì	Exp 2	Rep 3	45,4
siRNA Scramble + Ptac2S	Sì	Exp 2	Rep 4	45,3
siRNA Scramble + Ptac2S	Sì	Exp 2	Rep 5	46,0
siRNA Scramble + Ptac2S	Sì	Exp 2	Rep 6	45,8
siRNA Scramble + Ptac2S	Sì	Exp 2	Rep 7	42,5
siRNA Scramble + Ptac2S	Sì	Exp 2	Rep 8	50,0
siRNA Scramble + Ptac2S	Sì	Exp 3	Rep 1	48,1
siRNA Scramble + Ptac2S	Sì	Exp 3	Rep 2	46,6
siRNA Scramble + Ptac2S	Sì	Exp 3	Rep 3	46,5
siRNA Scramble + Ptac2S	Sì	Exp 3	Rep 4	43,8
siRNA Scramble + Ptac2S	Sì	Exp 3	Rep 5	49,0
siRNA Scramble + Ptac2S	Sì	Exp 3	Rep 6	43,9
siRNA Scramble + Ptac2S	Sì	Exp 3	Rep 7	48,4
siRNA Scramble + Ptac2S	Sì	Exp 3	Rep 8	48,6
siRNA Scramble + Ptac2S	Sì	Exp 4	Rep 1	46,9
siRNA Scramble + Ptac2S	Sì	Exp 4	Rep 2	42,5
siRNA Scramble + Ptac2S	Sì	Exp 4	Rep 3	42,8
siRNA Scramble + Ptac2S	Sì	Exp 4	Rep 4	48,8
siRNA Scramble + Ptac2S	Sì	Exp 4	Rep 5	50,9
siRNA Scramble + Ptac2S	Sì	Exp 4	Rep 6	51,6
siRNA Scramble + Ptac2S	Sì	Exp 4	Rep 7	48,8
siRNA Scramble + Ptac2S	Sì	Exp 4	Rep 8	45,9
siRNA Scramble + Ptac2S	Sì	Exp 5	Rep 1	41,4
siRNA Scramble + Ptac2S	Sì	Exp 5	Rep 2	43,6
siRNA Scramble + Ptac2S	Sì	Exp 5	Rep 3	44,2
siRNA Scramble + Ptac2S	Sì	Exp 5	Rep 4	45,3
siRNA Scramble + Ptac2S	Sì	Exp 5	Rep 5	43,9
siRNA Scramble + Ptac2S	Sì	Exp 5	Rep 6	48,8
siRNA Scramble + Ptac2S	Sì	Exp 5	Rep 7	49,1
siRNA Scramble + Ptac2S	Sì	Exp 5	Rep 8	47,5
siRNA PKC-δ + Ptac2S	Sì	Exp 1	Rep 1	77,9
siRNA PKC-δ + Ptac2S	Sì	Exp 1	Rep 2	78,8
siRNA PKC-δ + Ptac2S	Sì	Exp 1	Rep 3	83,5
siRNA PKC-δ + Ptac2S	Sì	Exp 1	Rep 4	76,8
siRNA PKC-δ + Ptac2S	Sì	Exp 1	Rep 5	77,0
siRNA PKC-δ + Ptac2S	Sì	Exp 1	Rep 6	78,6
siRNA PKC-δ + Ptac2S	Sì	Exp 1	Rep 7	74,2
siRNA PKC-δ + Ptac2S	Sì	Exp 1	Rep 8	72,7
siRNA PKC-δ + Ptac2S	Sì	Exp 2	Rep 1	72,9
siRNA PKC-δ + Ptac2S	Sì	Exp 2	Rep 2	72,5
siRNA PKC-δ + Ptac2S	Sì	Exp 2	Rep 3	73,1
siRNA PKC-δ + Ptac2S	Sì	Exp 2	Rep 4	77,6
siRNA PKC-δ + Ptac2S	Sì	Exp 2	Rep 5	77,3
siRNA PKC-δ + Ptac2S	Sì	Exp 2	Rep 6	74,7
siRNA PKC-δ + Ptac2S	Sì	Exp 2	Rep 7	76,0
siRNA PKC-δ + Ptac2S	Sì	Exp 2	Rep 8	80,7
siRNA PKC-δ + Ptac2S	Sì	Exp 3	Rep 1	76,1
siRNA PKC-δ + Ptac2S	Sì	Exp 3	Rep 2	78,0
siRNA PKC-δ + Ptac2S	Sì	Exp 3	Rep 3	80,7
siRNA PKC-δ + Ptac2S	Sì	Exp 3	Rep 4	77,7
siRNA PKC-δ + Ptac2S	Sì	Exp 3	Rep 5	76,0
siRNA PKC-δ + Ptac2S	Sì	Exp 3	Rep 6	73,2
siRNA PKC-δ + Ptac2S	Sì	Exp 3	Rep 7	77,8
siRNA PKC-δ + Ptac2S	Sì	Exp 3	Rep 8	79,1
siRNA PKC-δ + Ptac2S	Sì	Exp 4	Rep 1	78,8
siRNA PKC-δ + Ptac2S	Sì	Exp 4	Rep 2	78,0
siRNA PKC-δ + Ptac2S	Sì	Exp 4	Rep 3	79,3
siRNA PKC-δ + Ptac2S	Sì	Exp 4	Rep 4	79,2
siRNA PKC-δ + Ptac2S	Sì	Exp 4	Rep 5	75,3
siRNA PKC-δ + Ptac2S	Sì	Exp 4	Rep 6	79,6
siRNA PKC-δ + Ptac2S	Sì	Exp 4	Rep 7	75,3
siRNA PKC-δ + Ptac2S	Sì	Exp 4	Rep 8	73,8
siRNA PKC-δ + Ptac2S	Sì	Exp 5	Rep 1	76,4
siRNA PKC-δ + Ptac2S	Sì	Exp 5	Rep 2	82,2
siRNA PKC-δ + Ptac2S	Sì	Exp 5	Rep 3	77,7
siRNA PKC-δ + Ptac2S	Sì	Exp 5	Rep 4	78,6
siRNA PKC-δ + Ptac2S	Sì	Exp 5	Rep 5	76,7
siRNA PKC-δ + Ptac2S	Sì	Exp 5	Rep 6	78,4
siRNA PKC-δ + Ptac2S	Sì	Exp 5	Rep 7	77,0
siRNA PKC-δ + Ptac2S	Sì	Exp 5	Rep 8	75,0
siRNA PKC-ε + Ptac2S	Sì	Exp 1	Rep 1	41,8
siRNA PKC-ε + Ptac2S	Sì	Exp 1	Rep 2	45,2
siRNA PKC-ε + Ptac2S	Sì	Exp 1	Rep 3	44,0
siRNA PKC-ε + Ptac2S	Sì	Exp 1	Rep 4	44,8
siRNA PKC-ε + Ptac2S	Sì	Exp 1	Rep 5	51,1
siRNA PKC-ε + Ptac2S	Sì	Exp 1	Rep 6	46,4
siRNA PKC-ε + Ptac2S	Sì	Exp 1	Rep 7	44,0
siRNA PKC-ε + Ptac2S	Sì	Exp 1	Rep 8	41,7
siRNA PKC-ε + Ptac2S	Sì	Exp 2	Rep 1	45,3
siRNA PKC-ε + Ptac2S	Sì	Exp 2	Rep 2	49,3
siRNA PKC-ε + Ptac2S	Sì	Exp 2	Rep 3	49,5
siRNA PKC-ε + Ptac2S	Sì	Exp 2	Rep 4	44,5
siRNA PKC-ε + Ptac2S	Sì	Exp 2	Rep 5	39,2
siRNA PKC-ε + Ptac2S	Sì	Exp 2	Rep 6	47,1
siRNA PKC-ε + Ptac2S	Sì	Exp 2	Rep 7	46,4
siRNA PKC-ε + Ptac2S	Sì	Exp 2	Rep 8	45,3
siRNA PKC-ε + Ptac2S	Sì	Exp 3	Rep 1	43,0
siRNA PKC-ε + Ptac2S	Sì	Exp 3	Rep 2	45,3
siRNA PKC-ε + Ptac2S	Sì	Exp 3	Rep 3	43,8
siRNA PKC-ε + Ptac2S	Sì	Exp 3	Rep 4	43,9
siRNA PKC-ε + Ptac2S	Sì	Exp 3	Rep 5	44,7
siRNA PKC-ε + Ptac2S	Sì	Exp 3	Rep 6	40,2
siRNA PKC-ε + Ptac2S	Sì	Exp 3	Rep 7	48,1
siRNA PKC-ε + Ptac2S	Sì	Exp 3	Rep 8	46,1
siRNA PKC-ε + Ptac2S	Sì	Exp 4	Rep 1	42,0
siRNA PKC-ε + Ptac2S	Sì	Exp 4	Rep 2	41,5
siRNA PKC-ε + Ptac2S	Sì	Exp 4	Rep 3	48,4
siRNA PKC-ε + Ptac2S	Sì	Exp 4	Rep 4	43,7
siRNA PKC-ε + Ptac2S	Sì	Exp 4	Rep 5	42,3
siRNA PKC-ε + Ptac2S	Sì	Exp 4	Rep 6	47,4
siRNA PKC-ε + Ptac2S	Sì	Exp 4	Rep 7	46,5
siRNA PKC-ε + Ptac2S	Sì	Exp 4	Rep 8	47,2
siRNA PKC-ε + Ptac2S	Sì	Exp 5	Rep 1	44,6
siRNA PKC-ε + Ptac2S	Sì	Exp 5	Rep 2	46,5
siRNA PKC-ε + Ptac2S	Sì	Exp 5	Rep 3	45,7
siRNA PKC-ε + Ptac2S	Sì	Exp 5	Rep 4	48,9
siRNA PKC-ε + Ptac2S	Sì	Exp 5	Rep 5	46,2
siRNA PKC-ε + Ptac2S	Sì	Exp 5	Rep 6	45,8
siRNA PKC-ε + Ptac2S	Sì	Exp 5	Rep 7	45,3
siRNA PKC-ε + Ptac2S	Sì	Exp 5	Rep 8	46,1
Ptac2S da solo (Veicolo)	Sì	Exp 1	Rep 1	44,2
Ptac2S da solo (Veicolo)	Sì	Exp 1	Rep 2	45,4
Ptac2S da solo (Veicolo)	Sì	Exp 1	Rep 3	49,5
Ptac2S da solo (Veicolo)	Sì	Exp 1	Rep 4	45,7
Ptac2S da solo (Veicolo)	Sì	Exp 1	Rep 5	48,9
Ptac2S da solo (Veicolo)	Sì	Exp 1	Rep 6	47,7
Ptac2S da solo (Veicolo)	Sì	Exp 1	Rep 7	43,4
Ptac2S da solo (Veicolo)	Sì	Exp 1	Rep 8	46,9
Ptac2S da solo (Veicolo)	Sì	Exp 2	Rep 1	46,2
Ptac2S da solo (Veicolo)	Sì	Exp 2	Rep 2	47,9
Ptac2S da solo (Veicolo)	Sì	Exp 2	Rep 3	43,2
Ptac2S da solo (Veicolo)	Sì	Exp 2	Rep 4	44,8
Ptac2S da solo (Veicolo)	Sì	Exp 2	Rep 5	45,1
Ptac2S da solo (Veicolo)	Sì	Exp 2	Rep 6	53,0
Ptac2S da solo (Veicolo)	Sì	Exp 2	Rep 7	50,7
Ptac2S da solo (Veicolo)	Sì	Exp 2	Rep 8	46,2
Ptac2S da solo (Veicolo)	Sì	Exp 3	Rep 1	47,3
Ptac2S da solo (Veicolo)	Sì	Exp 3	Rep 2	47,3
Ptac2S da solo (Veicolo)	Sì	Exp 3	Rep 3	49,7
Ptac2S da solo (Veicolo)	Sì	Exp 3	Rep 4	47,3
Ptac2S da solo (Veicolo)	Sì	Exp 3	Rep 5	44,6
Ptac2S da solo (Veicolo)	Sì	Exp 3	Rep 6	38,8
Ptac2S da solo (Veicolo)	Sì	Exp 3	Rep 7	40,1
Ptac2S da solo (Veicolo)	Sì	Exp 3	Rep 8	41,7
Ptac2S da solo (Veicolo)	Sì	Exp 4	Rep 1	46,7
Ptac2S da solo (Veicolo)	Sì	Exp 4	Rep 2	46,8
Ptac2S da solo (Veicolo)	Sì	Exp 4	Rep 3	45,4
Ptac2S da solo (Veicolo)	Sì	Exp 4	Rep 4	49,1
Ptac2S da solo (Veicolo)	Sì	Exp 4	Rep 5	48,2
Ptac2S da solo (Veicolo)	Sì	Exp 4	Rep 6	46,0
Ptac2S da solo (Veicolo)	Sì	Exp 4	Rep 7	46,5
Ptac2S da solo (Veicolo)	Sì	Exp 4	Rep 8	50,1
Ptac2S da solo (Veicolo)	Sì	Exp 5	Rep 1	44,8
Ptac2S da solo (Veicolo)	Sì	Exp 5	Rep 2	45,6
Ptac2S da solo (Veicolo)	Sì	Exp 5	Rep 3	49,5
Ptac2S da solo (Veicolo)	Sì	Exp 5	Rep 4	41,7
Ptac2S da solo (Veicolo)	Sì	Exp 5	Rep 5	46,3
Ptac2S da solo (Veicolo)	Sì	Exp 5	Rep 6	40,3
Ptac2S da solo (Veicolo)	Sì	Exp 5	Rep 7	45,8
Ptac2S da solo (Veicolo)	Sì	Exp 5	Rep 8	46,7
SB203580 (15 µM) + Ptac2	Sì	Exp 1	Rep 1	69,9
SB203580 (15 µM) + Ptac2	Sì	Exp 1	Rep 2	65,6
SB203580 (15 µM) + Ptac2	Sì	Exp 1	Rep 3	67,9
SB203580 (15 µM) + Ptac2	Sì	Exp 1	Rep 4	68,9
SB203580 (15 µM) + Ptac2	Sì	Exp 1	Rep 5	68,9
SB203580 (15 µM) + Ptac2	Sì	Exp 1	Rep 6	69,9
SB203580 (15 µM) + Ptac2	Sì	Exp 1	Rep 7	72,4
SB203580 (15 µM) + Ptac2	Sì	Exp 1	Rep 8	64,9
SB203580 (15 µM) + Ptac2	Sì	Exp 2	Rep 1	69,1
SB203580 (15 µM) + Ptac2	Sì	Exp 2	Rep 2	67,3
SB203580 (15 µM) + Ptac2	Sì	Exp 2	Rep 3	69,4
SB203580 (15 µM) + Ptac2	Sì	Exp 2	Rep 4	67,1
SB203580 (15 µM) + Ptac2	Sì	Exp 2	Rep 5	68,3
SB203580 (15 µM) + Ptac2	Sì	Exp 2	Rep 6	68,9
SB203580 (15 µM) + Ptac2	Sì	Exp 2	Rep 7	70,1
SB203580 (15 µM) + Ptac2	Sì	Exp 2	Rep 8	68,1
SB203580 (15 µM) + Ptac2	Sì	Exp 3	Rep 1	66,4
SB203580 (15 µM) + Ptac2	Sì	Exp 3	Rep 2	65,1
SB203580 (15 µM) + Ptac2	Sì	Exp 3	Rep 3	67,4
SB203580 (15 µM) + Ptac2	Sì	Exp 3	Rep 4	66,1
SB203580 (15 µM) + Ptac2	Sì	Exp 3	Rep 5	70,1
SB203580 (15 µM) + Ptac2	Sì	Exp 3	Rep 6	62,2
SB203580 (15 µM) + Ptac2	Sì	Exp 3	Rep 7	62,8
SB203580 (15 µM) + Ptac2	Sì	Exp 3	Rep 8	63,3
SB203580 (15 µM) + Ptac2	Sì	Exp 4	Rep 1	73,2
SB203580 (15 µM) + Ptac2	Sì	Exp 4	Rep 2	70,7
SB203580 (15 µM) + Ptac2	Sì	Exp 4	Rep 3	72,7
SB203580 (15 µM) + Ptac2	Sì	Exp 4	Rep 4	71,5
SB203580 (15 µM) + Ptac2	Sì	Exp 4	Rep 5	65,7
SB203580 (15 µM) + Ptac2	Sì	Exp 4	Rep 6	73,2
SB203580 (15 µM) + Ptac2	Sì	Exp 4	Rep 7	75,7
SB203580 (15 µM) + Ptac2	Sì	Exp 4	Rep 8	71,2
SB203580 (15 µM) + Ptac2	Sì	Exp 5	Rep 1	66,6
SB203580 (15 µM) + Ptac2	Sì	Exp 5	Rep 2	66,5
SB203580 (15 µM) + Ptac2	Sì	Exp 5	Rep 3	72,1
SB203580 (15 µM) + Ptac2	Sì	Exp 5	Rep 4	72,1
SB203580 (15 µM) + Ptac2	Sì	Exp 5	Rep 5	69,6
SB203580 (15 µM) + Ptac2	Sì	Exp 5	Rep 6	61,2
SB203580 (15 µM) + Ptac2	Sì	Exp 5	Rep 7	68,1
SB203580 (15 µM) + Ptac2	Sì	Exp 5	Rep 8	70,3
SB203580 (30 µM) + Ptac2	Sì	Exp 1	Rep 1	85,4
SB203580 (30 µM) + Ptac2	Sì	Exp 1	Rep 2	83,4
SB203580 (30 µM) + Ptac2	Sì	Exp 1	Rep 3	90,6
SB203580 (30 µM) + Ptac2	Sì	Exp 1	Rep 4	84,9
SB203580 (30 µM) + Ptac2	Sì	Exp 1	Rep 5	84,8
SB203580 (30 µM) + Ptac2	Sì	Exp 1	Rep 6	83,5
SB203580 (30 µM) + Ptac2	Sì	Exp 1	Rep 7	81,7
SB203580 (30 µM) + Ptac2	Sì	Exp 1	Rep 8	88,8
SB203580 (30 µM) + Ptac2	Sì	Exp 2	Rep 1	78,0
SB203580 (30 µM) + Ptac2	Sì	Exp 2	Rep 2	85,3
SB203580 (30 µM) + Ptac2	Sì	Exp 2	Rep 3	79,5
SB203580 (30 µM) + Ptac2	Sì	Exp 2	Rep 4	87,6
SB203580 (30 µM) + Ptac2	Sì	Exp 2	Rep 5	88,3
SB203580 (30 µM) + Ptac2	Sì	Exp 2	Rep 6	85,8
SB203580 (30 µM) + Ptac2	Sì	Exp 2	Rep 7	84,7
SB203580 (30 µM) + Ptac2	Sì	Exp 2	Rep 8	84,3
SB203580 (30 µM) + Ptac2	Sì	Exp 3	Rep 1	84,5
SB203580 (30 µM) + Ptac2	Sì	Exp 3	Rep 2	89,6
SB203580 (30 µM) + Ptac2	Sì	Exp 3	Rep 3	84,2
SB203580 (30 µM) + Ptac2	Sì	Exp 3	Rep 4	84,3
SB203580 (30 µM) + Ptac2	Sì	Exp 3	Rep 5	87,5
SB203580 (30 µM) + Ptac2	Sì	Exp 3	Rep 6	84,6
SB203580 (30 µM) + Ptac2	Sì	Exp 3	Rep 7	87,6
SB203580 (30 µM) + Ptac2	Sì	Exp 3	Rep 8	87,9
SB203580 (30 µM) + Ptac2	Sì	Exp 4	Rep 1	87,3
SB203580 (30 µM) + Ptac2	Sì	Exp 4	Rep 2	86,2
SB203580 (30 µM) + Ptac2	Sì	Exp 4	Rep 3	86,2
SB203580 (30 µM) + Ptac2	Sì	Exp 4	Rep 4	85,3
SB203580 (30 µM) + Ptac2	Sì	Exp 4	Rep 5	85,6
SB203580 (30 µM) + Ptac2	Sì	Exp 4	Rep 6	80,9
SB203580 (30 µM) + Ptac2	Sì	Exp 4	Rep 7	85,4
SB203580 (30 µM) + Ptac2	Sì	Exp 4	Rep 8	77,9
SB203580 (30 µM) + Ptac2	Sì	Exp 5	Rep 1	88,2
SB203580 (30 µM) + Ptac2	Sì	Exp 5	Rep 2	86,0
SB203580 (30 µM) + Ptac2	Sì	Exp 5	Rep 3	87,5
SB203580 (30 µM) + Ptac2	Sì	Exp 5	Rep 4	79,1
SB203580 (30 µM) + Ptac2	Sì	Exp 5	Rep 5	82,7
SB203580 (30 µM) + Ptac2	Sì	Exp 5	Rep 6	80,0
SB203580 (30 µM) + Ptac2	Sì	Exp 5	Rep 7	88,3

ELABORAZIONE STATISTICA COMPLETA

Condizione Sperimentale	Ptac2S	Media (% del Ctrl)	Dev. Std. (S.D.)	Lettera Post-Hoc
Controllo non tra	No	100,9	2,2	a
siRNA Scramble	Sì	45,9	3,2	b
siRNA PKC-δ + I	Sì	77,1	3,2	c
siRNA PKC-ε + F	Sì	45,2	3,2	b
Ptac2S da solo (	Sì	46,1	3,2	b
SB203580 (15 µ	Sì	68,5	3,2	d
SB203580 (30 µ	Sì	85,0	3,2	c

fig 5 c

Condizione Sperimentale	Ptac2S (5 µM)	Esperimento	Replica Tecnica	Vitalità Cellulare (% del Ctrl)
Controllo non trattato (Ctrl NT)	No	Exp 1	Rep 1	102,5
Controllo non trattato (Ctrl NT)	No	Exp 1	Rep 2	106,5
Controllo non trattato (Ctrl NT)	No	Exp 1	Rep 3	102,8
Controllo non trattato (Ctrl NT)	No	Exp 1	Rep 4	99,0
Controllo non trattato (Ctrl NT)	No	Exp 1	Rep 5	99,4
Controllo non trattato (Ctrl NT)	No	Exp 1	Rep 6	98,8
Controllo non trattato (Ctrl NT)	No	Exp 1	Rep 7	100,7
Controllo non trattato (Ctrl NT)	No	Exp 1	Rep 8	102,4
Controllo non trattato (Ctrl NT)	No	Exp 2	Rep 1	102,3
Controllo non trattato (Ctrl NT)	No	Exp 2	Rep 2	107,3
Controllo non trattato (Ctrl NT)	No	Exp 2	Rep 3	102,2
Controllo non trattato (Ctrl NT)	No	Exp 2	Rep 4	101,4
Controllo non trattato (Ctrl NT)	No	Exp 2	Rep 5	104,4
Controllo non trattato (Ctrl NT)	No	Exp 2	Rep 6	103,0
Controllo non trattato (Ctrl NT)	No	Exp 2	Rep 7	98,8
Controllo non trattato (Ctrl NT)	No	Exp 2	Rep 8	102,8
Controllo non trattato (Ctrl NT)	No	Exp 3	Rep 1	97,7
Controllo non trattato (Ctrl NT)	No	Exp 3	Rep 2	97,4
Controllo non trattato (Ctrl NT)	No	Exp 3	Rep 3	100,1
Controllo non trattato (Ctrl NT)	No	Exp 3	Rep 4	102,0
Controllo non trattato (Ctrl NT)	No	Exp 3	Rep 5	99,0
Controllo non trattato (Ctrl NT)	No	Exp 3	Rep 6	100,5
Controllo non trattato (Ctrl NT)	No	Exp 3	Rep 7	101,1
Controllo non trattato (Ctrl NT)	No	Exp 3	Rep 8	95,9
Controllo non trattato (Ctrl NT)	No	Exp 4	Rep 1	102,5
Controllo non trattato (Ctrl NT)	No	Exp 4	Rep 2	98,9
Controllo non trattato (Ctrl NT)	No	Exp 4	Rep 3	98,6
Controllo non trattato (Ctrl NT)	No	Exp 4	Rep 4	102,4
Controllo non trattato (Ctrl NT)	No	Exp 4	Rep 5	97,5
Controllo non trattato (Ctrl NT)	No	Exp 4	Rep 6	102,7
Controllo non trattato (Ctrl NT)	No	Exp 4	Rep 7	103,5
Controllo non trattato (Ctrl NT)	No	Exp 4	Rep 8	100,7
Controllo non trattato (Ctrl NT)	No	Exp 5	Rep 1	99,8
Controllo non trattato (Ctrl NT)	No	Exp 5	Rep 2	100,6
Controllo non trattato (Ctrl NT)	No	Exp 5	Rep 3	100,0
Controllo non trattato (Ctrl NT)	No	Exp 5	Rep 4	99,8
Controllo non trattato (Ctrl NT)	No	Exp 5	Rep 5	98,8
Controllo non trattato (Ctrl NT)	No	Exp 5	Rep 6	99,5
Controllo non trattato (Ctrl NT)	No	Exp 5	Rep 7	101,7
Controllo non trattato (Ctrl NT)	No	Exp 5	Rep 8	100,5
siRNA Scramble + Ptac2S	Sì	Exp 1	Rep 1	46,6
siRNA Scramble + Ptac2S	Sì	Exp 1	Rep 2	47,4
siRNA Scramble + Ptac2S	Sì	Exp 1	Rep 3	44,3
siRNA Scramble + Ptac2S	Sì	Exp 1	Rep 4	42,5
siRNA Scramble + Ptac2S	Sì	Exp 1	Rep 5	43,2
siRNA Scramble + Ptac2S	Sì	Exp 1	Rep 6	42,6
siRNA Scramble + Ptac2S	Sì	Exp 1	Rep 7	40,1
siRNA Scramble + Ptac2S	Sì	Exp 1	Rep 8	44,7
siRNA Scramble + Ptac2S	Sì	Exp 2	Rep 1	47,9
siRNA Scramble + Ptac2S	Sì	Exp 2	Rep 2	43,0
siRNA Scramble + Ptac2S	Sì	Exp 2	Rep 3	45,4
siRNA Scramble + Ptac2S	Sì	Exp 2	Rep 4	45,3
siRNA Scramble + Ptac2S	Sì	Exp 2	Rep 5	46,0
siRNA Scramble + Ptac2S	Sì	Exp 2	Rep 6	45,8
siRNA Scramble + Ptac2S	Sì	Exp 2	Rep 7	42,5
siRNA Scramble + Ptac2S	Sì	Exp 2	Rep 8	50,0
siRNA Scramble + Ptac2S	Sì	Exp 3	Rep 1	48,1
siRNA Scramble + Ptac2S	Sì	Exp 3	Rep 2	46,6
siRNA Scramble + Ptac2S	Sì	Exp 3	Rep 3	46,5
siRNA Scramble + Ptac2S	Sì	Exp 3	Rep 4	43,8
siRNA Scramble + Ptac2S	Sì	Exp 3	Rep 5	49,0
siRNA Scramble + Ptac2S	Sì	Exp 3	Rep 6	43,9
siRNA Scramble + Ptac2S	Sì	Exp 3	Rep 7	48,4
siRNA Scramble + Ptac2S	Sì	Exp 3	Rep 8	48,6
siRNA Scramble + Ptac2S	Sì	Exp 4	Rep 1	46,9
siRNA Scramble + Ptac2S	Sì	Exp 4	Rep 2	42,5
siRNA Scramble + Ptac2S	Sì	Exp 4	Rep 3	42,8
siRNA Scramble + Ptac2S	Sì	Exp 4	Rep 4	48,8
siRNA Scramble + Ptac2S	Sì	Exp 4	Rep 5	50,9
siRNA Scramble + Ptac2S	Sì	Exp 4	Rep 6	51,6
siRNA Scramble + Ptac2S	Sì	Exp 4	Rep 7	48,8
siRNA Scramble + Ptac2S	Sì	Exp 4	Rep 8	45,9
siRNA Scramble + Ptac2S	Sì	Exp 5	Rep 1	41,4
siRNA Scramble + Ptac2S	Sì	Exp 5	Rep 2	43,6
siRNA Scramble + Ptac2S	Sì	Exp 5	Rep 3	44,2
siRNA Scramble + Ptac2S	Sì	Exp 5	Rep 4	45,3
siRNA Scramble + Ptac2S	Sì	Exp 5	Rep 5	43,9
siRNA Scramble + Ptac2S	Sì	Exp 5	Rep 6	48,8
siRNA Scramble + Ptac2S	Sì	Exp 5	Rep 7	49,1
siRNA Scramble + Ptac2S	Sì	Exp 5	Rep 8	47,5
siRNA PKC-δ + Ptac2S	Sì	Exp 1	Rep 1	77,9
siRNA PKC-δ + Ptac2S	Sì	Exp 1	Rep 2	78,8
siRNA PKC-δ + Ptac2S	Sì	Exp 1	Rep 3	83,5
siRNA PKC-δ + Ptac2S	Sì	Exp 1	Rep 4	76,8
siRNA PKC-δ + Ptac2S	Sì	Exp 1	Rep 5	77,0
siRNA PKC-δ + Ptac2S	Sì	Exp 1	Rep 6	78,6
siRNA PKC-δ + Ptac2S	Sì	Exp 1	Rep 7	74,2
siRNA PKC-δ + Ptac2S	Sì	Exp 1	Rep 8	72,7
siRNA PKC-δ + Ptac2S	Sì	Exp 2	Rep 1	72,9
siRNA PKC-δ + Ptac2S	Sì	Exp 2	Rep 2	72,5
siRNA PKC-δ + Ptac2S	Sì	Exp 2	Rep 3	73,1
siRNA PKC-δ + Ptac2S	Sì	Exp 2	Rep 4	77,6
siRNA PKC-δ + Ptac2S	Sì	Exp 2	Rep 5	77,3
siRNA PKC-δ + Ptac2S	Sì	Exp 2	Rep 6	74,7
siRNA PKC-δ + Ptac2S	Sì	Exp 2	Rep 7	76,0
siRNA PKC-δ + Ptac2S	Sì	Exp 2	Rep 8	80,7
siRNA PKC-δ + Ptac2S	Sì	Exp 3	Rep 1	76,1
siRNA PKC-δ + Ptac2S	Sì	Exp 3	Rep 2	78,0
siRNA PKC-δ + Ptac2S	Sì	Exp 3	Rep 3	80,7
siRNA PKC-δ + Ptac2S	Sì	Exp 3	Rep 4	77,7
siRNA PKC-δ + Ptac2S	Sì	Exp 3	Rep 5	76,0
siRNA PKC-δ + Ptac2S	Sì	Exp 3	Rep 6	73,2
siRNA PKC-δ + Ptac2S	Sì	Exp 3	Rep 7	77,8
siRNA PKC-δ + Ptac2S	Sì	Exp 3	Rep 8	79,1
siRNA PKC-δ + Ptac2S	Sì	Exp 4	Rep 1	78,8
siRNA PKC-δ + Ptac2S	Sì	Exp 4	Rep 2	78,0
siRNA PKC-δ + Ptac2S	Sì	Exp 4	Rep 3	79,3
siRNA PKC-δ + Ptac2S	Sì	Exp 4	Rep 4	79,2
siRNA PKC-δ + Ptac2S	Sì	Exp 4	Rep 5	75,3
siRNA PKC-δ + Ptac2S	Sì	Exp 4	Rep 6	79,6
siRNA PKC-δ + Ptac2S	Sì	Exp 4	Rep 7	75,3
siRNA PKC-δ + Ptac2S	Sì	Exp 4	Rep 8	73,8
siRNA PKC-δ + Ptac2S	Sì	Exp 5	Rep 1	76,4
siRNA PKC-δ + Ptac2S	Sì	Exp 5	Rep 2	82,2
siRNA PKC-δ + Ptac2S	Sì	Exp 5	Rep 3	77,7
siRNA PKC-δ + Ptac2S	Sì	Exp 5	Rep 4	78,6
siRNA PKC-δ + Ptac2S	Sì	Exp 5	Rep 5	76,7
siRNA PKC-δ + Ptac2S	Sì	Exp 5	Rep 6	78,4
siRNA PKC-δ + Ptac2S	Sì	Exp 5	Rep 7	77,0
siRNA PKC-δ + Ptac2S	Sì	Exp 5	Rep 8	75,0
siRNA PKC-ε + Ptac2S	Sì	Exp 1	Rep 1	41,8
siRNA PKC-ε + Ptac2S	Sì	Exp 1	Rep 2	45,2
siRNA PKC-ε + Ptac2S	Sì	Exp 1	Rep 3	44,0
siRNA PKC-ε + Ptac2S	Sì	Exp 1	Rep 4	44,8
siRNA PKC-ε + Ptac2S	Sì	Exp 1	Rep 5	51,1
siRNA PKC-ε + Ptac2S	Sì	Exp 1	Rep 6	46,4
siRNA PKC-ε + Ptac2S	Sì	Exp 1	Rep 7	44,0
siRNA PKC-ε + Ptac2S	Sì	Exp 1	Rep 8	41,7
siRNA PKC-ε + Ptac2S	Sì	Exp 2	Rep 1	45,3
siRNA PKC-ε + Ptac2S	Sì	Exp 2	Rep 2	49,3
siRNA PKC-ε + Ptac2S	Sì	Exp 2	Rep 3	49,5
siRNA PKC-ε + Ptac2S	Sì	Exp 2	Rep 4	44,5
siRNA PKC-ε + Ptac2S	Sì	Exp 2	Rep 5	39,2
siRNA PKC-ε + Ptac2S	Sì	Exp 2	Rep 6	47,1
siRNA PKC-ε + Ptac2S	Sì	Exp 2	Rep 7	46,4
siRNA PKC-ε + Ptac2S	Sì	Exp 2	Rep 8	45,3
siRNA PKC-ε + Ptac2S	Sì	Exp 3	Rep 1	43,0
siRNA PKC-ε + Ptac2S	Sì	Exp 3	Rep 2	45,3
siRNA PKC-ε + Ptac2S	Sì	Exp 3	Rep 3	43,8
siRNA PKC-ε + Ptac2S	Sì	Exp 3	Rep 4	43,9
siRNA PKC-ε + Ptac2S	Sì	Exp 3	Rep 5	44,7
siRNA PKC-ε + Ptac2S	Sì	Exp 3	Rep 6	40,2
siRNA PKC-ε + Ptac2S	Sì	Exp 3	Rep 7	48,1
siRNA PKC-ε + Ptac2S	Sì	Exp 3	Rep 8	46,1
siRNA PKC-ε + Ptac2S	Sì	Exp 4	Rep 1	42,0
siRNA PKC-ε + Ptac2S	Sì	Exp 4	Rep 2	41,5
siRNA PKC-ε + Ptac2S	Sì	Exp 4	Rep 3	48,4
siRNA PKC-ε + Ptac2S	Sì	Exp 4	Rep 4	43,7
siRNA PKC-ε + Ptac2S	Sì	Exp 4	Rep 5	42,3
siRNA PKC-ε + Ptac2S	Sì	Exp 4	Rep 6	47,4
siRNA PKC-ε + Ptac2S	Sì	Exp 4	Rep 7	46,5
siRNA PKC-ε + Ptac2S	Sì	Exp 4	Rep 8	47,2
siRNA PKC-ε + Ptac2S	Sì	Exp 5	Rep 1	44,6
siRNA PKC-ε + Ptac2S	Sì	Exp 5	Rep 2	46,5
siRNA PKC-ε + Ptac2S	Sì	Exp 5	Rep 3	45,7
siRNA PKC-ε + Ptac2S	Sì	Exp 5	Rep 4	48,9
siRNA PKC-ε + Ptac2S	Sì	Exp 5	Rep 5	46,2
siRNA PKC-ε + Ptac2S	Sì	Exp 5	Rep 6	45,8
siRNA PKC-ε + Ptac2S	Sì	Exp 5	Rep 7	45,3
siRNA PKC-ε + Ptac2S	Sì	Exp 5	Rep 8	46,1
Ptac2S da solo (Veicolo)	Sì	Exp 1	Rep 1	44,2
Ptac2S da solo (Veicolo)	Sì	Exp 1	Rep 2	45,4
Ptac2S da solo (Veicolo)	Sì	Exp 1	Rep 3	49,5
Ptac2S da solo (Veicolo)	Sì	Exp 1	Rep 4	45,7
Ptac2S da solo (Veicolo)	Sì	Exp 1	Rep 5	48,9
Ptac2S da solo (Veicolo)	Sì	Exp 1	Rep 6	47,7
Ptac2S da solo (Veicolo)	Sì	Exp 1	Rep 7	43,4
Ptac2S da solo (Veicolo)	Sì	Exp 1	Rep 8	46,9
Ptac2S da solo (Veicolo)	Sì	Exp 2	Rep 1	46,2
Ptac2S da solo (Veicolo)	Sì	Exp 2	Rep 2	47,9
Ptac2S da solo (Veicolo)	Sì	Exp 2	Rep 3	43,2
Ptac2S da solo (Veicolo)	Sì	Exp 2	Rep 4	44,8
Ptac2S da solo (Veicolo)	Sì	Exp 2	Rep 5	45,1
Ptac2S da solo (Veicolo)	Sì	Exp 2	Rep 6	53,0
Ptac2S da solo (Veicolo)	Sì	Exp 2	Rep 7	50,7
Ptac2S da solo (Veicolo)	Sì	Exp 2	Rep 8	46,2
Ptac2S da solo (Veicolo)	Sì	Exp 3	Rep 1	47,3
Ptac2S da solo (Veicolo)	Sì	Exp 3	Rep 2	47,3
Ptac2S da solo (Veicolo)	Sì	Exp 3	Rep 3	49,7
Ptac2S da solo (Veicolo)	Sì	Exp 3	Rep 4	47,3
Ptac2S da solo (Veicolo)	Sì	Exp 3	Rep 5	44,6
Ptac2S da solo (Veicolo)	Sì	Exp 3	Rep 6	38,8
Ptac2S da solo (Veicolo)	Sì	Exp 3	Rep 7	40,1
Ptac2S da solo (Veicolo)	Sì	Exp 3	Rep 8	41,7
Ptac2S da solo (Veicolo)	Sì	Exp 4	Rep 1	46,7
Ptac2S da solo (Veicolo)	Sì	Exp 4	Rep 2	46,8
Ptac2S da solo (Veicolo)	Sì	Exp 4	Rep 3	45,4
Ptac2S da solo (Veicolo)	Sì	Exp 4	Rep 4	49,1
Ptac2S da solo (Veicolo)	Sì	Exp 4	Rep 5	48,2
Ptac2S da solo (Veicolo)	Sì	Exp 4	Rep 6	46,0
Ptac2S da solo (Veicolo)	Sì	Exp 4	Rep 7	46,5
Ptac2S da solo (Veicolo)	Sì	Exp 4	Rep 8	50,1
Ptac2S da solo (Veicolo)	Sì	Exp 5	Rep 1	44,8
Ptac2S da solo (Veicolo)	Sì	Exp 5	Rep 2	45,6
Ptac2S da solo (Veicolo)	Sì	Exp 5	Rep 3	49,5
Ptac2S da solo (Veicolo)	Sì	Exp 5	Rep 4	41,7
Ptac2S da solo (Veicolo)	Sì	Exp 5	Rep 5	46,3
Ptac2S da solo (Veicolo)	Sì	Exp 5	Rep 6	40,3
Ptac2S da solo (Veicolo)	Sì	Exp 5	Rep 7	45,8
Ptac2S da solo (Veicolo)	Sì	Exp 5	Rep 8	46,7
SB203580 (15 µM) + Ptac2S	Sì	Exp 1	Rep 1	69,9
SB203580 (15 µM) + Ptac2S	Sì	Exp 1	Rep 2	65,6
SB203580 (15 µM) + Ptac2S	Sì	Exp 1	Rep 3	67,9
SB203580 (15 µM) + Ptac2S	Sì	Exp 1	Rep 4	68,9
SB203580 (15 µM) + Ptac2S	Sì	Exp 1	Rep 5	68,9
SB203580 (15 µM) + Ptac2S	Sì	Exp 1	Rep 6	69,9
SB203580 (15 µM) + Ptac2S	Sì	Exp 1	Rep 7	72,4
SB203580 (15 µM) + Ptac2S	Sì	Exp 1	Rep 8	64,9
SB203580 (15 µM) + Ptac2S	Sì	Exp 2	Rep 1	69,1
SB203580 (15 µM) + Ptac2S	Sì	Exp 2	Rep 2	67,3
SB203580 (15 µM) + Ptac2S	Sì	Exp 2	Rep 3	69,4
SB203580 (15 µM) + Ptac2S	Sì	Exp 2	Rep 4	67,1
SB203580 (15 µM) + Ptac2S	Sì	Exp 2	Rep 5	68,3
SB203580 (15 µM) + Ptac2S	Sì	Exp 2	Rep 6	68,9
SB203580 (15 µM) + Ptac2S	Sì	Exp 2	Rep 7	70,1
SB203580 (15 µM) + Ptac2S	Sì	Exp 2	Rep 8	68,1
SB203580 (15 µM) + Ptac2S	Sì	Exp 3	Rep 1	66,4
SB203580 (15 µM) + Ptac2S	Sì	Exp 3	Rep 2	65,1
SB203580 (15 µM) + Ptac2S	Sì	Exp 3	Rep 3	67,4
SB203580 (15 µM) + Ptac2S	Sì	Exp 3	Rep 4	66,1
SB203580 (15 µM) + Ptac2S	Sì	Exp 3	Rep 5	70,1
SB203580 (15 µM) + Ptac2S	Sì	Exp 3	Rep 6	62,2
SB203580 (15 µM) + Ptac2S	Sì	Exp 3	Rep 7	62,8
SB203580 (15 µM) + Ptac2S	Sì	Exp 3	Rep 8	63,3
SB203580 (15 µM) + Ptac2S	Sì	Exp 4	Rep 1	73,2
SB203580 (15 µM) + Ptac2S	Sì	Exp 4	Rep 2	70,7
SB203580 (15 µM) + Ptac2S	Sì	Exp 4	Rep 3	72,7
SB203580 (15 µM) + Ptac2S	Sì	Exp 4	Rep 4	71,5
SB203580 (15 µM) + Ptac2S	Sì	Exp 4	Rep 5	65,7
SB203580 (15 µM) + Ptac2S	Sì	Exp 4	Rep 6	73,2
SB203580 (15 µM) + Ptac2S	Sì	Exp 4	Rep 7	75,7
SB203580 (15 µM) + Ptac2S	Sì	Exp 4	Rep 8	71,2
SB203580 (15 µM) + Ptac2S	Sì	Exp 5	Rep 1	66,6
SB203580 (15 µM) + Ptac2S	Sì	Exp 5	Rep 2	66,5
SB203580 (15 µM) + Ptac2S	Sì	Exp 5	Rep 3	72,1
SB203580 (15 µM) + Ptac2S	Sì	Exp 5	Rep 4	72,1
SB203580 (15 µM) + Ptac2S	Sì	Exp 5	Rep 5	69,6
SB203580 (15 µM) + Ptac2S	Sì	Exp 5	Rep 6	61,2
SB203580 (15 µM) + Ptac2S	Sì	Exp 5	Rep 7	68,1
SB203580 (15 µM) + Ptac2S	Sì	Exp 5	Rep 8	70,3
SB203580 (30 µM) + Ptac2S	Sì	Exp 1	Rep 1	85,4
SB203580 (30 µM) + Ptac2S	Sì	Exp 1	Rep 2	83,4
SB203580 (30 µM) + Ptac2S	Sì	Exp 1	Rep 3	90,6
SB203580 (30 µM) + Ptac2S	Sì	Exp 1	Rep 4	84,9
SB203580 (30 µM) + Ptac2S	Sì	Exp 1	Rep 5	84,8
SB203580 (30 µM) + Ptac2S	Sì	Exp 1	Rep 6	83,5
SB203580 (30 µM) + Ptac2S	Sì	Exp 1	Rep 7	81,7
SB203580 (30 µM) + Ptac2S	Sì	Exp 1	Rep 8	88,8
SB203580 (30 µM) + Ptac2S	Sì	Exp 2	Rep 1	78,0
SB203580 (30 µM) + Ptac2S	Sì	Exp 2	Rep 2	85,3
SB203580 (30 µM) + Ptac2S	Sì	Exp 2	Rep 3	79,5
SB203580 (30 µM) + Ptac2S	Sì	Exp 2	Rep 4	87,6
SB203580 (30 µM) + Ptac2S	Sì	Exp 2	Rep 5	88,3
SB203580 (30 µM) + Ptac2S	Sì	Exp 2	Rep 6	85,8
SB203580 (30 µM) + Ptac2S	Sì	Exp 2	Rep 7	84,7
SB203580 (30 µM) + Ptac2S	Sì	Exp 2	Rep 8	84,3
SB203580 (30 µM) + Ptac2S	Sì	Exp 3	Rep 1	84,5
SB203580 (30 µM) + Ptac2S	Sì	Exp 3	Rep 2	89,6
SB203580 (30 µM) + Ptac2S	Sì	Exp 3	Rep 3	84,2
SB203580 (30 µM) + Ptac2S	Sì	Exp 3	Rep 4	84,3
SB203580 (30 µM) + Ptac2S	Sì	Exp 3	Rep 5	87,5
SB203580 (30 µM) + Ptac2S	Sì	Exp 3	Rep 6	84,6
SB203580 (30 µM) + Ptac2S	Sì	Exp 3	Rep 7	87,6
SB203580 (30 µM) + Ptac2S	Sì	Exp 3	Rep 8	87,9
SB203580 (30 µM) + Ptac2S	Sì	Exp 4	Rep 1	87,3
SB203580 (30 µM) + Ptac2S	Sì	Exp 4	Rep 2	86,2
SB203580 (30 µM) + Ptac2S	Sì	Exp 4	Rep 3	86,2
SB203580 (30 µM) + Ptac2S	Sì	Exp 4	Rep 4	85,3
SB203580 (30 µM) + Ptac2S	Sì	Exp 4	Rep 5	85,6
SB203580 (30 µM) + Ptac2S	Sì	Exp 4	Rep 6	80,9
SB203580 (30 µM) + Ptac2S	Sì	Exp 4	Rep 7	85,4
SB203580 (30 µM) + Ptac2S	Sì	Exp 4	Rep 8	77,9
SB203580 (30 µM) + Ptac2S	Sì	Exp 5	Rep 1	88,2
SB203580 (30 µM) + Ptac2S	Sì	Exp 5	Rep 2	86,0
SB203580 (30 µM) + Ptac2S	Sì	Exp 5	Rep 3	87,5
SB203580 (30 µM) + Ptac2S	Sì	Exp 5	Rep 4	79,1
SB203580 (30 µM) + Ptac2S	Sì	Exp 5	Rep 5	82,7
SB203580 (30 µM) + Ptac2S	Sì	Exp 5	Rep 6	80,0
SB203580 (30 µM) + Ptac2S	Sì	Exp 5	Rep 7	88,3
SB203580 (30 µM) + Ptac2S	Sì	Exp 5	Rep 8	82,2

ELABORAZIONE STATISTICA COMPLETA (Formule Attive)					
Condizione Sperimentale	Ptac2S	Media (% del Ctrl)	Dev. Std. (S.D.)	Errore Standard (S.E.M.)	Lettera Post-Hoc
Controllo non trattato (Ctrl NT)	No	100,9	2,2	0,2	a
siRNA Scramble + Ptac2S	Sì	45,9	3,2	0,2	b
siRNA PKC-δ + Ptac2S	Sì	77,1	3,2	0,2	c
siRNA PKC-ε + Ptac2S	Sì	45,2	3,2	0,2	b
Ptac2S da solo (Veicolo)	Sì	46,1	3,2	0,2	b
SB203580 (15 µM) + Ptac2S	Sì	68,5	3,2	1,2	d
SB203580 (30 µM) + Ptac2S	Sì	84,9	3,2	0,2	c

VOLUME TUMORE

Gruppo	Settimana	Topo	Volume Tumorale (mm³)
Saline	0	Topo 1	46,4
Saline	0	Topo 2	45,6
Saline	0	Topo 3	56,3
Saline	0	Topo 4	43,4
Saline	0	Topo 5	45,5
Saline	0	Topo 6	50,1
Saline	0	Topo 7	52,4
Saline	0	Topo 8	50,7
Saline	1	Topo 1	180,2
Saline	1	Topo 2	202,5
Saline	1	Topo 3	175,9
Saline	1	Topo 4	172,8
Saline	1	Topo 5	186,2
Saline	1	Topo 6	144,5
Saline	1	Topo 7	168,4
Saline	1	Topo 8	185,3
Saline	2	Topo 1	237,5
Saline	2	Topo 2	208,8
Saline	2	Topo 3	224,7
Saline	2	Topo 4	221,4
Saline	2	Topo 5	235,2
Saline	2	Topo 6	186,3
Saline	2	Topo 7	229,7
Saline	2	Topo 8	175,9
Saline	3	Topo 1	205,7
Saline	3	Topo 2	278,2
Saline	3	Topo 3	267,0
Saline	3	Topo 4	331,5
Saline	3	Topo 5	323,9
Saline	3	Topo 6	256,1
Saline	3	Topo 7	330,5
Saline	3	Topo 8	263,9
Saline	4	Topo 1	395,9
Saline	4	Topo 2	385,9
Saline	4	Topo 3	405,5
Saline	4	Topo 4	439,3
Saline	4	Topo 5	430,6
Saline	4	Topo 6	416,8
Saline	4	Topo 7	431,2
Saline	4	Topo 8	423,0
Saline	5	Topo 1	467,0
Saline	5	Topo 2	461,5
Saline	5	Topo 3	476,5
Saline	5	Topo 4	535,3
Saline	5	Topo 5	489,8
Saline	5	Topo 6	646,5
Saline	5	Topo 7	455,4
Saline	5	Topo 8	438,4
Cisplatin 10mg/Kg	0	Topo 1	44,6
Cisplatin 10mg/Kg	0	Topo 2	48,1
Cisplatin 10mg/Kg	0	Topo 3	50,9
Cisplatin 10mg/Kg	0	Topo 4	53,0
Cisplatin 10mg/Kg	0	Topo 5	45,7
Cisplatin 10mg/Kg	0	Topo 6	42,5
Cisplatin 10mg/Kg	0	Topo 7	45,4
Cisplatin 10mg/Kg	0	Topo 8	57,1
Cisplatin 10mg/Kg	1	Topo 1	160,2
Cisplatin 10mg/Kg	1	Topo 2	139,9
Cisplatin 10mg/Kg	1	Topo 3	140,7
Cisplatin 10mg/Kg	1	Topo 4	136,3
Cisplatin 10mg/Kg	1	Topo 5	165,7
Cisplatin 10mg/Kg	1	Topo 6	140,6
Cisplatin 10mg/Kg	1	Topo 7	148,8
Cisplatin 10mg/Kg	1	Topo 8	162,4
Cisplatin 10mg/Kg	2	Topo 1	165,7
Cisplatin 10mg/Kg	2	Topo 2	174,2
Cisplatin 10mg/Kg	2	Topo 3	143,5
Cisplatin 10mg/Kg	2	Topo 4	158,6
Cisplatin 10mg/Kg	2	Topo 5	168,8
Cisplatin 10mg/Kg	2	Topo 6	188,2
Cisplatin 10mg/Kg	2	Topo 7	203,6
Cisplatin 10mg/Kg	2	Topo 8	179,6
Cisplatin 10mg/Kg	3	Topo 1	241,8
Cisplatin 10mg/Kg	3	Topo 2	239,3
Cisplatin 10mg/Kg	3	Topo 3	263,0
Cisplatin 10mg/Kg	3	Topo 4	258,1
Cisplatin 10mg/Kg	3	Topo 5	242,1
Cisplatin 10mg/Kg	3	Topo 6	208,9
Cisplatin 10mg/Kg	3	Topo 7	258,4
Cisplatin 10mg/Kg	3	Topo 8	244,9
Cisplatin 10mg/Kg	4	Topo 1	340,5
Cisplatin 10mg/Kg	4	Topo 2	299,0
Cisplatin 10mg/Kg	4	Topo 3	364,5
Cisplatin 10mg/Kg	4	Topo 4	288,2
Cisplatin 10mg/Kg	4	Topo 5	352,6
Cisplatin 10mg/Kg	4	Topo 6	303,8
Cisplatin 10mg/Kg	4	Topo 7	283,0
Cisplatin 10mg/Kg	4	Topo 8	262,8
Cisplatin 10mg/Kg	5	Topo 1	398,3
Cisplatin 10mg/Kg	5	Topo 2	468,4
Cisplatin 10mg/Kg	5	Topo 3	441,4
Cisplatin 10mg/Kg	5	Topo 4	435,7
Cisplatin 10mg/Kg	5	Topo 5	299,2
Cisplatin 10mg/Kg	5	Topo 6	436,7
Cisplatin 10mg/Kg	5	Topo 7	429,8
Cisplatin 10mg/Kg	5	Topo 8	380,5
Ptac2S 10mg/Kg	0	Topo 1	56,6
Ptac2S 10mg/Kg	0	Topo 2	55,9
Ptac2S 10mg/Kg	0	Topo 3	51,7
Ptac2S 10mg/Kg	0	Topo 4	44,4
Ptac2S 10mg/Kg	0	Topo 5	54,5
Ptac2S 10mg/Kg	0	Topo 6	50,5
Ptac2S 10mg/Kg	0	Topo 7	42,3
Ptac2S 10mg/Kg	0	Topo 8	56,9
Ptac2S 10mg/Kg	1	Topo 1	100,1
Ptac2S 10mg/Kg	1	Topo 2	70,6
Ptac2S 10mg/Kg	1	Topo 3	83,7
Ptac2S 10mg/Kg	1	Topo 4	88,6
Ptac2S 10mg/Kg	1	Topo 5	104,3
Ptac2S 10mg/Kg	1	Topo 6	66,4
Ptac2S 10mg/Kg	1	Topo 7	95,9
Ptac2S 10mg/Kg	1	Topo 8	91,5
Ptac2S 10mg/Kg	2	Topo 1	119,9
Ptac2S 10mg/Kg	2	Topo 2	109,3
Ptac2S 10mg/Kg	2	Topo 3	100,7
Ptac2S 10mg/Kg	2	Topo 4	93,2
Ptac2S 10mg/Kg	2	Topo 5	83,8
Ptac2S 10mg/Kg	2	Topo 6	102,5
Ptac2S 10mg/Kg	2	Topo 7	97,5
Ptac2S 10mg/Kg	2	Topo 8	126,3
Ptac2S 10mg/Kg	3	Topo 1	110,5
Ptac2S 10mg/Kg	3	Topo 2	125,0
Ptac2S 10mg/Kg	3	Topo 3	95,5
Ptac2S 10mg/Kg	3	Topo 4	113,8
Ptac2S 10mg/Kg	3	Topo 5	124,1
Ptac2S 10mg/Kg	3	Topo 6	132,9
Ptac2S 10mg/Kg	3	Topo 7	142,6
Ptac2S 10mg/Kg	3	Topo 8	119,3
Ptac2S 10mg/Kg	4	Topo 1	111,1
Ptac2S 10mg/Kg	4	Topo 2	137,7
Ptac2S 10mg/Kg	4	Topo 3	138,7
Ptac2S 10mg/Kg	4	Topo 4	138,3
Ptac2S 10mg/Kg	4	Topo 5	118,2
Ptac2S 10mg/Kg	4	Topo 6	149,2
Ptac2S 10mg/Kg	4	Topo 7	131,5
Ptac2S 10mg/Kg	4	Topo 8	141,8
Ptac2S 10mg/Kg	5	Topo 1	227,3
Ptac2S 10mg/Kg	5	Topo 2	210,4
Ptac2S 10mg/Kg	5	Topo 3	202,3
Ptac2S 10mg/Kg	5	Topo 4	249,6
Ptac2S 10mg/Kg	5	Topo 5	201,2
Ptac2S 10mg/Kg	5	Topo 6	187,5
Ptac2S 10mg/Kg	5	Topo 7	197,8
Ptac2S 10mg/Kg	5	Topo 8	232,6

STATISTICA DESCRITTIVA - VOLUME TUMORALE (mm³)				
Gruppo	Settimana	Media (Formula)	Dev. Std. (S.D.)	Significatività
Saline	0	48,8	4,3	
Saline	1	177,0	16,7	
Saline	2	214,9	22,9	
Saline	3	282,1	44,1	
Saline	4	416,0	18,7	
Saline	5	496,3	67,2	
Cisplatin 10mg/Kg	0	48,4	4,9	
Cisplatin 10mg/Kg	1	149,3	11,8	
Cisplatin 10mg/Kg	2	172,8	18,4	
Cisplatin 10mg/Kg	3	244,6	17,0	
Cisplatin 10mg/Kg	4	311,8	36,4	
Cisplatin 10mg/Kg	5	411,3	52,7	*
Ptac2S 10mg/Kg	0	51,6	5,6	
Ptac2S 10mg/Kg	1	87,6	13,5	* #
Ptac2S 10mg/Kg	2	104,2	13,9	* #
Ptac2S 10mg/Kg	3	120,5	14,4	* #
Ptac2S 10mg/Kg	4	133,3	12,7	* #
Ptac2S 10mg/Kg	5	213,6	20,9	* #

PESO CORPOREO

Gruppo	Settimana	Topo	Peso Corporeo (g)
Saline	0	Topo 1	24,2
Saline	0	Topo 2	23,4
Saline	0	Topo 3	23,9
Saline	0	Topo 4	22,5
Saline	0	Topo 5	23,0
Saline	0	Topo 6	23,1
Saline	0	Topo 7	24,4
Saline	0	Topo 8	22,2
Saline	1	Topo 1	23,9
Saline	1	Topo 2	25,4
Saline	1	Topo 3	25,4
Saline	1	Topo 4	24,1
Saline	1	Topo 5	25,3
Saline	1	Topo 6	26,1
Saline	1	Topo 7	23,9
Saline	1	Topo 8	25,3
Saline	2	Topo 1	26,9
Saline	2	Topo 2	29,9
Saline	2	Topo 3	24,4
Saline	2	Topo 4	27,8
Saline	2	Topo 5	26,5
Saline	2	Topo 6	26,8
Saline	2	Topo 7	29,5
Saline	2	Topo 8	26,9
Saline	3	Topo 1	31,7
Saline	3	Topo 2	27,9
Saline	3	Topo 3	29,0
Saline	3	Topo 4	27,8
Saline	3	Topo 5	27,5
Saline	3	Topo 6	28,6
Saline	3	Topo 7	28,1
Saline	3	Topo 8	28,8
Saline	4	Topo 1	25,3
Saline	4	Topo 2	31,1
Saline	4	Topo 3	28,1
Saline	4	Topo 4	30,8
Saline	4	Topo 5	26,9
Saline	4	Topo 6	28,7
Saline	4	Topo 7	32,8
Saline	4	Topo 8	27,1
Saline	5	Topo 1	26,9
Saline	5	Topo 2	28,3
Saline	5	Topo 3	29,8
Saline	5	Topo 4	30,1
Saline	5	Topo 5	31,0
Saline	5	Topo 6	28,7
Saline	5	Topo 7	26,8
Saline	5	Topo 8	28,5
Cisplatin 10mg/Kg	0	Topo 1	23,2
Cisplatin 10mg/Kg	0	Topo 2	24,0
Cisplatin 10mg/Kg	0	Topo 3	22,2
Cisplatin 10mg/Kg	0	Topo 4	22,4
Cisplatin 10mg/Kg	0	Topo 5	22,6
Cisplatin 10mg/Kg	0	Topo 6	22,3
Cisplatin 10mg/Kg	0	Topo 7	23,4
Cisplatin 10mg/Kg	0	Topo 8	23,5
Cisplatin 10mg/Kg	1	Topo 1	20,6
Cisplatin 10mg/Kg	1	Topo 2	23,0
Cisplatin 10mg/Kg	1	Topo 3	19,0
Cisplatin 10mg/Kg	1	Topo 4	21,7
Cisplatin 10mg/Kg	1	Topo 5	22,7
Cisplatin 10mg/Kg	1	Topo 6	24,1
Cisplatin 10mg/Kg	1	Topo 7	22,2
Cisplatin 10mg/Kg	1	Topo 8	23,4
Cisplatin 10mg/Kg	2	Topo 1	22,3
Cisplatin 10mg/Kg	2	Topo 2	21,8
Cisplatin 10mg/Kg	2	Topo 3	21,9
Cisplatin 10mg/Kg	2	Topo 4	24,9
Cisplatin 10mg/Kg	2	Topo 5	24,2
Cisplatin 10mg/Kg	2	Topo 6	23,5
Cisplatin 10mg/Kg	2	Topo 7	27,4
Cisplatin 10mg/Kg	2	Topo 8	22,8
Cisplatin 10mg/Kg	3	Topo 1	24,8
Cisplatin 10mg/Kg	3	Topo 2	24,3
Cisplatin 10mg/Kg	3	Topo 3	23,6
Cisplatin 10mg/Kg	3	Topo 4	27,2
Cisplatin 10mg/Kg	3	Topo 5	26,1
Cisplatin 10mg/Kg	3	Topo 6	21,9
Cisplatin 10mg/Kg	3	Topo 7	23,3
Cisplatin 10mg/Kg	3	Topo 8	24,5
Cisplatin 10mg/Kg	4	Topo 1	25,7
Cisplatin 10mg/Kg	4	Topo 2	22,5
Cisplatin 10mg/Kg	4	Topo 3	21,2
Cisplatin 10mg/Kg	4	Topo 4	21,7
Cisplatin 10mg/Kg	4	Topo 5	24,4
Cisplatin 10mg/Kg	4	Topo 6	24,3
Cisplatin 10mg/Kg	4	Topo 7	25,0
Cisplatin 10mg/Kg	4	Topo 8	23,4
Cisplatin 10mg/Kg	5	Topo 1	23,4
Cisplatin 10mg/Kg	5	Topo 2	22,2
Cisplatin 10mg/Kg	5	Topo 3	25,8
Cisplatin 10mg/Kg	5	Topo 4	25,9
Cisplatin 10mg/Kg	5	Topo 5	26,8
Cisplatin 10mg/Kg	5	Topo 6	26,5
Cisplatin 10mg/Kg	5	Topo 7	25,0
Cisplatin 10mg/Kg	5	Topo 8	27,3
Ptac2S 10mg/Kg	0	Topo 1	23,5
Ptac2S 10mg/Kg	0	Topo 2	22,6
Ptac2S 10mg/Kg	0	Topo 3	24,0
Ptac2S 10mg/Kg	0	Topo 4	22,2
Ptac2S 10mg/Kg	0	Topo 5	22,0
Ptac2S 10mg/Kg	0	Topo 6	23,9
Ptac2S 10mg/Kg	0	Topo 7	23,8
Ptac2S 10mg/Kg	0	Topo 8	23,3
Ptac2S 10mg/Kg	1	Topo 1	24,9
Ptac2S 10mg/Kg	1	Topo 2	25,2
Ptac2S 10mg/Kg	1	Topo 3	27,4
Ptac2S 10mg/Kg	1	Topo 4	26,3
Ptac2S 10mg/Kg	1	Topo 5	26,4
Ptac2S 10mg/Kg	1	Topo 6	24,2
Ptac2S 10mg/Kg	1	Topo 7	25,9
Ptac2S 10mg/Kg	1	Topo 8	23,7
Ptac2S 10mg/Kg	2	Topo 1	27,9
Ptac2S 10mg/Kg	2	Topo 2	28,1
Ptac2S 10mg/Kg	2	Topo 3	26,4
Ptac2S 10mg/Kg	2	Topo 4	30,2
Ptac2S 10mg/Kg	2	Topo 5	28,3
Ptac2S 10mg/Kg	2	Topo 6	24,9
Ptac2S 10mg/Kg	2	Topo 7	28,3
Ptac2S 10mg/Kg	2	Topo 8	26,9
Ptac2S 10mg/Kg	3	Topo 1	28,6
Ptac2S 10mg/Kg	3	Topo 2	29,3
Ptac2S 10mg/Kg	3	Topo 3	29,2
Ptac2S 10mg/Kg	3	Topo 4	25,7
Ptac2S 10mg/Kg	3	Topo 5	26,9
Ptac2S 10mg/Kg	3	Topo 6	29,7
Ptac2S 10mg/Kg	3	Topo 7	30,5
Ptac2S 10mg/Kg	3	Topo 8	31,3
Ptac2S 10mg/Kg	4	Topo 1	31,6
Ptac2S 10mg/Kg	4	Topo 2	26,0
Ptac2S 10mg/Kg	4	Topo 3	30,1
Ptac2S 10mg/Kg	4	Topo 4	26,2
Ptac2S 10mg/Kg	4	Topo 5	31,3
Ptac2S 10mg/Kg	4	Topo 6	29,4
Ptac2S 10mg/Kg	4	Topo 7	27,3
Ptac2S 10mg/Kg	4	Topo 8	31,9
Ptac2S 10mg/Kg	5	Topo 1	29,9
Ptac2S 10mg/Kg	5	Topo 2	26,3
Ptac2S 10mg/Kg	5	Topo 3	29,4
Ptac2S 10mg/Kg	5	Topo 4	27,9
Ptac2S 10mg/Kg	5	Topo 5	29,2
Ptac2S 10mg/Kg	5	Topo 6	28,3
Ptac2S 10mg/Kg	5	Topo 7	31,0
Ptac2S 10mg/Kg	5	Topo 8	26,9

STATISTICA DESCRITTIVA - PESO CORPOREO (g)				
Gruppo	Settimana	Media (Formula)	Dev. Std. (S.D.)	Significatività
Saline	0	23,3	0,8	
Saline	1	24,9	0,8	
Saline	2	27,3	1,8	
Saline	3	28,7	1,3	
Saline	4	28,9	2,5	
Saline	5	28,8	1,5	
Cisplatin 10mg/Kg	0	23,0	0,7	
Cisplatin 10mg/Kg	1	22,1	1,6	
Cisplatin 10mg/Kg	2	23,6	1,9	*
Cisplatin 10mg/Kg	3	24,5	1,6	*
Cisplatin 10mg/Kg	4	23,5	1,6	*
Cisplatin 10mg/Kg	5	25,4	1,8	*
Ptac2S 10mg/Kg	0	23,2	0,8	
Ptac2S 10mg/Kg	1	25,5	1,2	
Ptac2S 10mg/Kg	2	27,6	1,6	
Ptac2S 10mg/Kg	3	28,9	1,8	
Ptac2S 10mg/Kg	4	29,2	2,4	
Ptac2S 10mg/Kg	5	28,6	1,6	
